# Carrier strategies boost the application of CRISPR/Cas system in gene therapy

**DOI:** 10.1002/EXP.20210081

**Published:** 2022-03-15

**Authors:** Zunkai Xu, Qingnan Wang, Haiping Zhong, Yaoyao Jiang, Xiaoguang Shi, Bo Yuan, Na Yu, Shubiao Zhang, Xiaoyong Yuan, Shutao Guo, Yang Yang

**Affiliations:** ^1^ Key Laboratory of Functional Polymer Materials of Ministry of Education State Key Laboratory of Medicinal Chemical Biology and Institute of Polymer Chemistry College of Chemistry Nankai University Tianjin China; ^2^ State Key Laboratory of Biotherapy and Cancer Center West China Hospital Sichuan University and Collaborative Innovation Center Chengdu China; ^3^ School of Medicine Nankai University Tianjin China; ^4^ Tianjin Key Laboratory of Ophthalmology and Visual Science Tianjin Eye Institute Tianjin Eye Hospital Tianjin China; ^5^ Translational Medicine Center Key Laboratory of Molecular Target & Clinical Pharmacology School of Pharmaceutical Sciences and The Second Affiliated Hospital Guangzhou Medical University Guangzhou China; ^6^ Key Laboratory of Biotechnology and Bioresources Utilization of Ministry of Education Dalian Minzu University Dalian China; ^7^ Clinical College of Ophthalmology Tianjin Medical University Tianjin China

**Keywords:** CRISPR, drug delivery, gene therapy, genome editing, nanomaterials

## Abstract

Emerging clustered regularly interspaced short palindromic repeat/associated protein (CRISPR/Cas) genome editing technology shows great potential in gene therapy. However, proteins and nucleic acids suffer from enzymatic degradation in the physiological environment and low permeability into cells. Exploiting carriers to protect the CRISPR system from degradation, enhance its targeting of specific tissues and cells, and reduce its immunogenicity is essential to stimulate its clinical applications. Here, the authors review the state‐of‐the‐art CRISPR delivery systems and their applications, and describe strategies to improve the safety and efficacy of CRISPR mediated genome editing, categorized by three types of cargo formats, that is, Cas: single‐guide RNA ribonucleoprotein, Cas mRNA and single‐guide RNA, and Cas plasmid expressing CRISPR/Cas systems. The authors hope this review will help develop safe and efficient nanomaterial‐based carriers for CRISPR tools.

## Graphical abstract

This review mainly focuses on the state‐of‐the‐art CRISPR delivery systems and their applications, categorized by three types of cargo formats, that is, Cas: single‐guide RNA ribonucleoprotein, Cas mRNA and single‐guide RNA, and Cas plasmid expressing CRISPR/Cas systems.

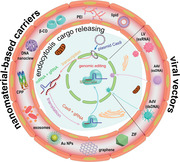

## 1 INTRODUCTION

In the past several decades, gene therapy has shown tremendous potential in treating human diseases like cancers, infectious diseases, genopathy, etc.^[^ ^1^ ^]^ Although traditional gene therapy strategies can rehabilitate the function of missing genes by transfecting plasmid DNA or messenger RNA (mRNA) and silence target genes by using RNA interference (RNAi) technologies, they cannot eradicate pathogenic mutations and have a risk of disease recurrence.^[^ ^2^ ^]^ A more effective strategy is to employ genome editing technologies that could precisely disrupt, insert, or replace a DNA sequence in the genome. Genome editing technologies, such as zinc‐finger nucleases (ZFNs) and transcription activator‐like effector nucleases (TALENs), evolved rapidly in the last few decades and showed great promise in gene therapy.^[^ ^3^ ^]^ However, for ZFNs and TALENs, the DNA‐binding domains are complex proteins, requiring sophisticated design and optimization, limiting the practical applications to some extent (Table 1).

**TABLE 1:** Characteristics of CRISPR/Cas9, TALENs, and ZFNs

	CRISPR/Cas9^[^ ^4^ ^]^	TALENs^[^ ^5^ ^]^	ZFNs^[^ ^6^ ^]^
Mechanism	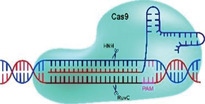	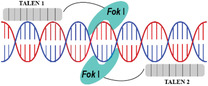	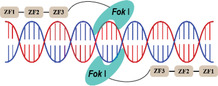
Cleavage domain	Cas9 endonuclease	Fok l endonuclease	Fok l endonuclease
Binding domain	Complex of Cas9 endonuclease and guide RNA	Transcription activator‐like effector nucleases	Zinc‐finger protein repeats
Target specificity	DNA‐RNA complementation in the guide of 20 bp targeting region of guide RNA	Each TALE protein unit recognizes 1 bp DNA	Each zinc‐finger protein recognizes 3 bp DNA
Editing efficiency	High	High	Low
Ease of engineering	Easy	Moderate	Difficult

The genome‐editing field has been significantly advanced with the recruitment of CRISPR associated protein 9 (Cas9) in mammalian cells in 2013.^[^ ^7^ ^]^ In the CRISPR/Cas9 system, guide RNA and Cas9 protein are the main working elements, eliminating the design of complex protein domains required for ZFNs and TALENs. Guide RNA will guide the Cas9 endonuclease to the specific double‐stranded DNA sequence to execute the editing, enabling targeted gene knockout and donor gene knockin. This disruptive guide RNA‐DNA recognition can significantly simplify the editing.^[^ ^8^ ^]^ In 2016, the Lu group launched the first clinical trial to assess the safety of PD‐1 knockout T cells in treating non‐small cell lung cancer (ClinicalTrials.gov Identifier: NCT02793856). Moreover, the CRISPR has been tested to correct frequent IVS26 CEP290 mutation for treating Leber congenital amaurosis type 10 (ClinicalTrials.gov Identifier: NCT03872479). However, developing safe and efficient CRISPR/Cas9 delivery methodologies remains challenging.

There are three common methods in the CRISPR/Cas9 delivery, including physical method (such as electroporation and microinjection), viral vectors (such as lentivirus (LVs), adenovirus (AVs), and adeno‐associated virus (AAVs)), and nanomaterial‐based carriers (such as lipid nanoparticles (LNPs)). Although electroporation^[^ ^9^ ^]^ and microinjection^[^ ^10^ ^]^ are efficient in mammalian cells and can be used to transfer immune/stem cells in vitro for adoptive cell therapy using CRISPR/Cas9, physical operation methods often destroy cells and are not amenable to be employed in vivo. In contrast, viral vectors, which display efficient transportation and stable gene expression in vivo, are the most studied gene delivery tools in clinical trials.^[^ ^11^ ^]^ Nevertheless, problems associated with viral vectors, including immunogenic adverse response, limited packaging capacity, and insertional mutagenesis, significantly hinder their broad applications. Alternatively, non‐viral carriers (such as nanomaterials), which have good biocompatibility and multi‐functionality and can protect cargos from degradation in the bloodstream, developed rapidly and could be safe alternatives to viral vectors.^[^ ^12^ ^]^ So far, three kinds of CRISPR cargo formats, that is, ribonucleoprotein (RNP) complex of Cas9 protein and sgRNA, Cas9 mRNA and sgRNA, and plasmid of Cas9/sgRNA, have been delivered into the mammalian cells by nanomaterials. However, the clinical application of CRISPR by non‐viral delivery systems is remarkedly hampered by the low transfection efficiency and non‐specific delivery. Improving the efficiency of existing vectors and developing more efficient non‐viral nanomaterial‐based delivery systems remain one mainstream to boost CRISPR/Cas9 clinical transformation.^[^ ^13^ ^]^

Although viral vectors and nanomaterial‐based carriers have been extensively studied in nucleic acid delivery, requirements for developing efficient CRISPR/Cas delivery systems are distinct because of different cargo formats (Cas9 RNP, Cas9 mRNA/sgRNA, and plasmid Cas9).^[^ ^14^ ^]^ Moving CRISPR/Cas genome editing tools forward, the advances in delivery systems of CRISPR/Cas should be urgently updated.^[^ ^15^ ^]^ Here, we summarize the recent development of viral vectors and nanomaterial‐based carriers in CRISPR/Cas delivery systems to stimulate innovations of carrier design to fulfill clinical needs. Given the adverse immunogenicity, low efficacy of homology directed repair (HDR), and other limitations in CRISPR, this review also analyzes the tactics to overcome these problems.

## 2 CRISPR/CAS SYSTEMS AND CARRIER‐BASED DELIVERY STRATEGIES

### 2.1 CRISPR/Cas systems

**FIGURE 1:**
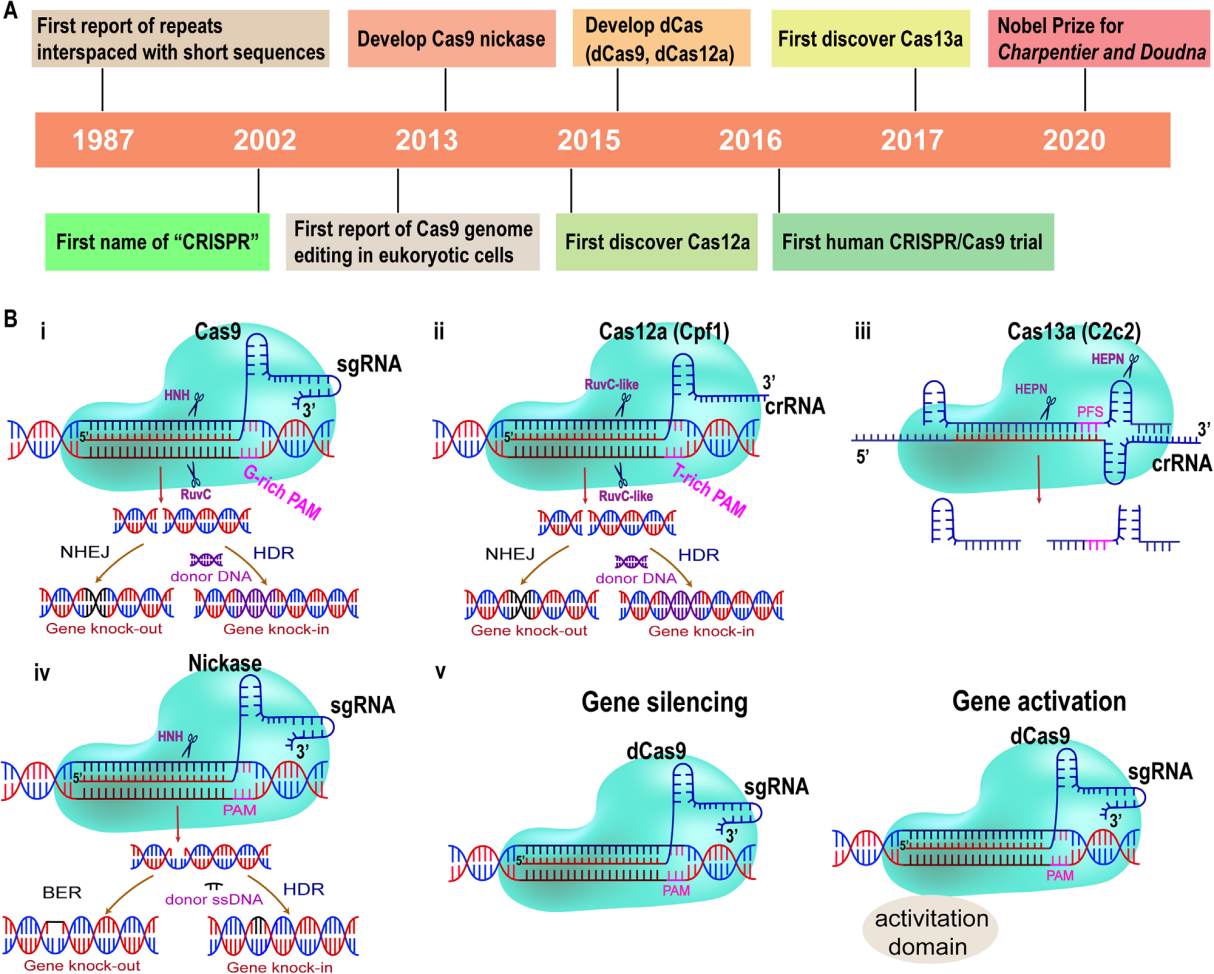
(A) Key events in the history of CRISPR systems; (B) mechanism of class 2 CRISPR/Cas genome editing systems. (i) Cas9 endonuclease is directed to a specific genomic locus by sgRNA and G‐rich PAM sequence, and then the HNH and RuvC domain of the endonuclease cut the double‐stranded DNA. DNA DSBs will be repaired by NHEJ or HDR (with the donor DNA) mechanisms. (ii) Cas12a (Cpf1) endonuclease is directed to a specific genomic locus by crRNA and a T‐rich PAM sequence (TTTV), and then RuvC‐like domains of the endonuclease cut the double‐stranded DNA. DNA DSBs will be repaired by NHEJ or HDR (with the donor DNA) mechanisms. (iii) Cas13a (C2c2) nuclease is directed to a specific genomic locus by crRNA and protospacer flanking sites (PFS) sequence (3’ A, U, or C (not required by all orthologs)), and then HEPN domains of the endonuclease cut the single‐stranded RNA (ssRNA). (iv) Cas9 nickase with point mutations at HNH or RuvC (not shown above) domain can bind to the target site and cleave a single strand of DNA. DNA single‐strand break (SSBs) will be repaired by base excision repair (BER) or HDR (with the donor ssDNA) mechanisms. (v) The dCas9 variants can bind DNA but cannot cleave it because of the mutation of endonuclease domains, which can be used for gene silencing or activation

It took decades for the development of CRISPR‐based gene therapy (Figure 1A).^[^ ^16^ ^]^ So far, three classes of genome editing mechanisms in CRISPR (class 1–3) have been identified. Site‐specific Cas protein and non‐coding CRISPR‐RNA (crRNA) are major components of the CRISPR system. Class 2 CRISPR is much simpler in engineering than class 1 and 3, as the class 2 CRISPR locus utilizes a sole Cas9 protein to create site‐specific double stranded DNA breaks (DSBs) in targeted sequence. In the CRISPR/Cas9 system, crRNA first recognizes a protospacer‐adjacent motif (PAM) sequence (e.g., 5′‐NGG, usually containing 3–5 nucleotide) in the genome and then matches with a complementary 20‐nt genomic sequence. Next, the trans‐activating crRNA (tracrRNA) will pair with crRNA by the repeat sequence and bind the Cas9 endonuclease by double‐stranded structure, forming the Cas9/crRNA/tracrRNA complex. The HNH and RuvC domains of Cas9 endonuclease are then cut from 3‐nucleotide of complementary DNA strand and non‐complementary DNA strand at the upstream of PAM site to achieve the site‐specific DSBs. The tracrRNA and crRNA can be engineered as a single guide RNA (sgRNA) with the 20‐nucleotide sequence at the 5′ end and the double‐stranded structure at the 3′ end, which will create a simpler sgRNA/Cas9 system to locate the dsDNA sequence.^[^ ^17^ ^]^ DSBs can be repaired by non‐homologous end joining (NHEJ) or HDR mechanisms (Figures 1B, 1i). NHEJ can generate frameshift mutation through random gene insertion or deletion, resulting in the permanent gene knockout. When DNA templates with homologous regions on both sides exist, HDR can integrate them into the DSB sites to achieve precise gene modification.^[^ ^18^ ^]^ In 2015, the class 2 type V endonuclease‐Cas12a (formerly known as Cpf1), which contains smaller size endonuclease (mRNA format: ≈1.3 kb), short guide RNA matching (42–44 nt), and T (thymine)‐rich recognized PAMs, was discovered and complements Cas9‐editing (Figure 1B, 1ii).^[^ ^19^ ^]^ In 2017, Zhang and colleagues discovered Cas13a (or C2c2) and grouped it in class 2 type VI CRISPR system (Figures 1B, 1iii). Cas13a nuclease only cleaves single‐strand RNA (ssRNA) containing protospacer flanking sites (PFS) sequence in the guidance with crRNA (28‐nt guides), and thus further expand the applications of CRISPR‐based genome editing.^[^ ^20^ ^]^ Mutated endonucleases were also designed in type II CRISPR systems. For example, Cas9 nickase, which retains HNH or RuvC domain, could precisely induce single‐strand nicks, regulating single‐stranded DNA (ssDNA) specific editing and prioritizing the HDR repair pathway (Figures 1B, 1iv).^[^ ^21^ ^]^ Moreover, Endonuclease Dead Cas (dCas), including dCas9 and dCas12a, whose endonuclease domains are point‐mutated, are also developed. Such dCas cannot induce sequence breakage but maintain the binding activity of the targeted site and silence or activate the gene expression (Figures 1B, 1v).^[^ ^22^ ^]^ More details on class 1 and class 3 CRISPR/Cas systems, as well as the repair mechanisms (NHEJ and HDR), have been reviewed elsewhere.^[^ ^23^ ^]^

### 2.2 Carriers‐based delivery strategies

**FIGURE 2:**
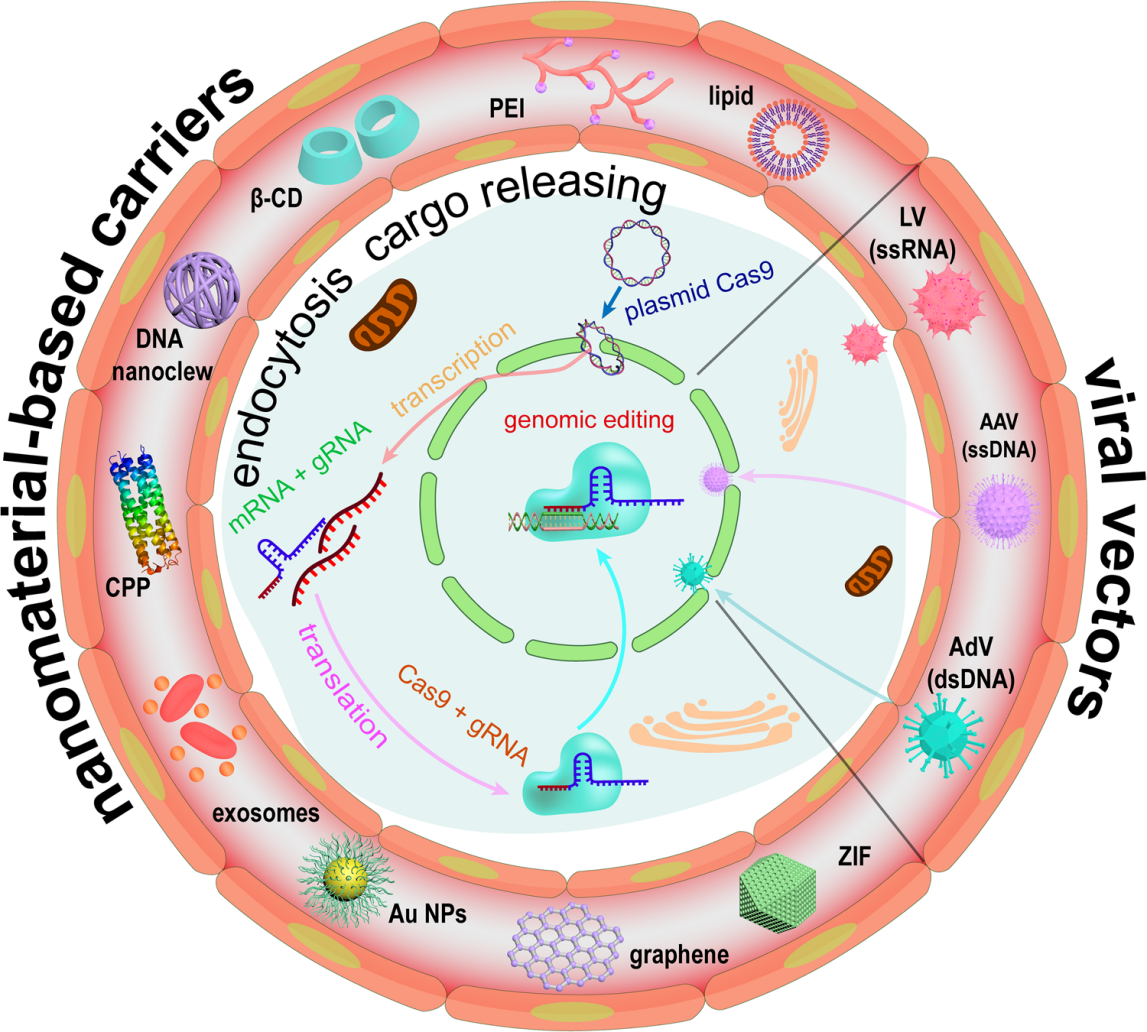
Overview of the delivery and expression of CRISPR/Cas systems in vivo

Proteins and nucleic acids suffer from enzymatic degradation in the physiological environment and low permeability into cells. Carriers are usually employed to solve these problems. In this review, we will focus on the delivery strategies for CRISPR/Cas9 systems. There are two major carrier strategies to deliver CRISPR/Cas9 systems: viral delivery strategy and nanomaterial‐based delivery strategy (Figure 2 and Table 2). The selection of delivery systems requires consideration of cargos, editing efficiency, insertion mutation, off‐target effect, and immunogenicity based on downstream applications. Viral vectors are commonly used to transfer genes for gene augmentation therapy, taking the inherent advantage of tropism.^[^ ^28^ ^]^ Improved production and safety of viral vectors have been achieved during the past decade.^[^ ^29^ ^]^ LVs, AVs and AAVs have been extensively investigated in preclinical models and clinical trials for genome editing and have shown superior transfection efficacy. However, pre‐existing immunity and insertional mutagenesis of viral vectors limited the clinic transformations of gene therapy.^[^ ^30^ ^]^ With the advantages of low cost, ease of manufacturing, low immunogenicity, and no limitation in the size of transgenic DNA, nanomaterial‐based carriers have also been enormously studied for gene therapy. Most importantly, transient transfection of DNA plasmids, mRNA, and RNP by nanomaterial‐based carriers is highly advantageous to minimize non‐specific outcomes of the CRISPR system. Advances and barriers in viral vectors and the nanomaterial‐based carriers used to deliver Cas9 RNP, Cas9 mRNA/sgRNA, and pCas9 will be introduced in detail in Sections 3, 4, 5, 6.

**TABLE 2:** Delivery strategies of CRISPR/Cas9

	Schematic diagram	Characters	Advantages	Disadvantages	Mechanisms
Viral genome expressing Cas9 and sgRNA^[^ ^24^ ^]^	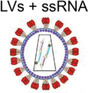	< 8.5 kb; 80–120 nm	Large packaging capacity	Potential insertional mutagenesis	Two copies of positive‐strand RNA containing gene are encapsulated by the protein capsid and envelope in LVs.
	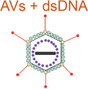	8–30 kb; 80–100 nm	Large packaging capacity; Transient protein expression	High immunogenicity	Double strand genome DNA containing Cas9 gene are encapsulated by the protein capsid and envelope in AVs.
	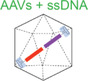	< 4.5 kb; 20–22 nm	Broad tropism; Low immunogenicity	Low packaging capacity	A single‐stranded genome containing Cas9 gene is encapsulated by the protein capsid in AAVs.
Cas9/sgRNA delivered by nanomaterial‐based delivery systems^[^ ^25^ ^]^	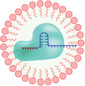	Cas9: ≈160 kDa	Transient duration; Low off‐target effects	Difficult for delivery; High cost	The Cas9:sgRNA RNP can be obtained by electrostatic interaction and turns to be negatively charged and directly works in the genome.
mRNA/sgRNA delivered by nanomaterial‐based delivery systems^[^ ^26^ ^]^	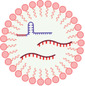	mRNA: 4500 nt	Transient expression; Low off‐target effects	Poor stability	Cas9 mRNA and sgRNA can be co‐delivered, and Cas9 mRNA can be translated in the cytoplasm.
Plasmid Cas9 delivered by nanomaterial‐based delivery systems^15^, ^27^ ^]^	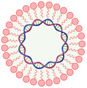	spCas9: ≈4.2 kb Plasmid: > 9.0 kb;	Good stability; Cost‐effective	Integration risk; Low efficiency	Cas9 mRNA and sgRNA can be expressed by the same pCas9 in the nucleus.

## 3 VIRAL VECTORS

### 3.1 LVs

LVs are a subclass of retroviral vectors that can integrate viral DNA into targeted cells. They are derived from HIV and other LVs. To minimize the revertant replication, these vectors are transformed with <5% of the HIV genome, and <25% of their genome consists of packing structures.^[24c^ ^]^ Because of the integration capability, exogenous genes could be efficiently transferred into host cells and stably expressed in targeted cells and their progenies.^[24b^ ^]^ In addition, LVs can carry larger and more complex gene cassettes.^[^ ^31^ ^]^ For example, carrying a catalytically inactive Cas9 (dCas9) fusion of Tet1 or Dnmt3a enabled DNA methylation at targeted gene sites and finally realized epigenetic regulation.^[^ ^32^ ^]^ It's necessary to identify regulating factors of transcription in human pluripotent stem cells. dCas9 fused with an effector domain could interrogate cellular differentiation course.^[^ ^33^ ^]^ LVs are the appropriate carriers in the genome editing of engineered T cells. Specifically knocking out endogenous TCR in T cells and simultaneously changing into a cancer‐reactive receptor of choice lead to improved efficiencies of cancer immunotherapies.^[^ ^34^ ^]^ Gene disruption in virtue of LVs is a feasible way to evaluate gene function. Knocking out by CRISPR/Cas9 LVs, UTX and UTY were confirmed as inhibitors of urothelial bladder cancer.^[^ ^35^ ^]^ KRAS is frequently mutated in many kinds of human tumors. Specifically targeting and disrupting mutant KRAS alleles through encoding Cas9 by the LVs in KRAS‐mutant cancer cells, cell proliferation was inhibited in vivo. While these Cas9 and sgRNA did not change cell survival in cells containing wild‐type KRAS. It provided supports that lentiviral delivery of Cas9 could be applied to locate oncogene mutations.^[^ ^36^ ^]^ Through introducing sgRNA and Cas9 into mouse HSC, up to five genes could be modified simultaneously, thereby leading to myeloid malignancy. This should be useful to constructing in vivo cancer models and reflect genetic complexity in human diseases.^[^ ^37^ ^]^ To establish a convenient system to estimate disease attributes of different clonal hematopoiesis driver genes, the Kenneth group applied LVs loaded with Cas9 to inactivate DNA Dnmt3a. They found that inactivating DNMT3A mutations in hematopoietic cells would lead to cardiovascular disease.^[^ ^38^ ^]^ Furthermore, some researchers took advantage of LVs‐mediated genome editing technology in multiple fields, including the regulation of transcriptional activation, against human immunodeficiency virus (HIV) infection, essential genes screening, and preventing the accumulation of off‐targets.^[^ ^39^ ^]^ For avoiding the genomic integration risk, integrase‐defective lentiviral vectors (IDLVs) had been utilized to express ZFNs and donor templates in vitro. In the form of episome, the IDLVs genome would gradually vanish by dilution in dividing cells as a result of short‐lived genome editing elements.^[^ ^40^ ^]^

### 3.2 AVs

As an unintegrated virus, AVs are vectors that can produce a number of recombinant viruses in infected differentiated and non‐dividing cells. Undeveloped AVs contain a 36 kb genome. They specifically tend to transduce pulmonary epithelial cells. For this reason, human AVs were initially used in treating cystic fibrosis. In engineered AVs, replacing viral proteins with therapeutic elements makes it possible to propagate exogenetic genes in a variety of cells.^[^ ^41^ ^]^ HSPC gene therapy is the major treatment for many hereditary disorders. Aimed at simplifying HSPC gene therapy, investigators successfully disrupted a BCL11A binding site, a repressor binding region within the γ‐globin promoter, induced by adenoviral vector expressing Cas9 and finally achieved γ‐globin reactivation. This study verified that it's possible to edit the HSPC genome by CRISPR‐Cas9 in vivo.^[^ ^42^ ^]^ In NSCLC cases, nearly 15% occur mutations in the EGFR gene. Co‐expression of EGFR mutation‐targeted guide RNA and Streptococcus pyogenes Cas9 (SpCas9) via adenoviral vector would specifically destroy the oncogenic mutation and result in the inhibition of cancer cells growth.^[^ ^43^ ^]^ In cancer therapy, specifically targeting genomic rearrangements is still an unattainable goal. By delivering nickase Cas9^D10A^ mediated suicide‐gene insertion at genome rearrangement site through AVs, animals showed decreased tumor burden. The AVs‐mediated genome editing approach showed therapeutic possibilities for human cancers carrying aberrant fusion genes.^[^ ^44^ ^]^ When AVs‐mediated therapeutic genome editing targeted the SERPINA1 gene in hepatocytes, it would relieve liver damage in the α1‐antitrypsin deficiency mouse model.^[^ ^45^ ^]^ A system based on AVs was verified to recognize the off‐target of CRISPR, thereby providing a blueprint to develop therapeutic genome editing. By utilizing high‐capacity adenoviral vectors, one vector delivered multiplexed sgRNA expression cassettes could target and degrade the hepatitis B virus genome.^[^ ^46^ ^]^ Recently, there was a big breakthrough for monogenic lung disease therapy. Researchers achieved gene editing in utero by Avs delivering CRISPR elements for inactivating mutant genes and improved lung morphology in fetuses and postnatal mice. This approach doubtlessly provided a promising treatment for monogenic diseases that were lethal at birth.^[^ ^47^ ^]^

### 3.3 AAVs

AAVs have been used as gene carriers for three decades. Different serotypes of AAVs can be taken for different target tissues. The recombinant AAVs enclose the therapeutic gene of interest under the control of an appropriate promoter for treatment needs. After infecting the host cell, the genetic material of recombinant AAVs persist mainly as an episomal form. Even though the permanent integration will not occur, the viral DNA could stably, and long term express in non‐dividing cells. Moreover, AAVs are not associated with any known disease.^[^ ^48^ ^]^ Over the past few years, AAVs gene therapy has exhibited good performance in clinical trials of several diseases.^[^ ^49^ ^]^ Recently, the FDA approved the first AAVs gene augmentation therapy for an inherited disease.^[^ ^50^ ^]^ Preliminary results from an AAVs‐ZFNs mediated genome editing clinical trial recently showed success in editing patients’ DNA. Combining CRISPR with AAVs also increase the feasibility of genome editing. For genome editing, we constructed a dual AAVs expressing CRISPR/Cas9 and donor DNA to correct gene mutation in newborn ornithine transcarbamylase deficiency (OTCD) mouse model. The results showed that mutations were corrected in 10% of hepatocytes and increased survival in mice challenged with a high‐protein diet. This study proved the efficacy of genetic mutation disease following in vivo genome editing induced by AAVs delivery vehicles.^[^ ^51^ ^]^ Furthermore, we developed a dual AAVs‐CRISPR‐Cas9‐mediated gene‐targeting approach that could be broadly applied for sustained treatment of patients with hemophilia B and restored hemostasis in both neonatal and adult mouse models.^[^ ^52^ ^]^ In the deltaE50‐MD dog model of DMD, Amoasii et al. showed that AAVs‐CRISPR‐Cas9 could restore the dystrophin level from 3% to 90%. These large animal data suggested that AAVs‐mediated CRISPR could support clinical value in the DMD treatment.^[^ ^53^ ^]^

Aimed at establishing a novel avenue for AAVs‐based therapy, the Belmonte group devised a homology‐independent targeted integration approach and demonstrated its efficacy of improving visual function in a rat model of the retinal degeneration condition retinitis pigmentosa.^[^ ^54^ ^]^ For remitting retinitis pigmentosa, Yu et al. utilized the AAVs‐CRISPR system to knock down Nrl gene expression and preserved cone function in retinal degeneration mouse models.^[^ ^55^ ^]^ Another study focused on overcoming the mutations in rhodopsin by AAVs‐CRISPR induced genome surgery and renovated autosomal dominant retinitis pigmentosa.^[^ ^56^ ^]^ Ran et al. packaged SaCas9 and sgRNA into AAVs to target PCSK9, and the gene modification efficiency was over 40% in liver hepatocytes.^[^ ^57^ ^]^ Recently, through a PAM variant of Cas9, AAVs‐mediated editing could selectively destroy the mutant allele of Tmc1, preventing deafness in Beethoven mice. The specific disruption could also be achieved in DFNA36 human cell line. Based on these results, some other mutations might be targeted by the strategy.^[^ ^58^ ^]^ However, a long‐term study revealed that AAVs‐CRISPR could induce unintended genome and transcript alterations, which is of particular concern.^[^ ^59^ ^]^

Despite the remarkable progress in viral vector‐mediated genome editing in the field of gene therapy, these approaches still have some limitations. LVs will randomly integrate into host cells for stable expression, which might increase the risk of mutagenesis and oncogenesis.^[^ ^60^ ^]^ As unintegrated viruses, AVs and AAVs will be diluted in dividing cells and suffer from pre‐existing immunity of the host. The loss of therapeutic episomal vectors may decrease the efficiency of genome editing.^[^ ^61^ ^]^ Once the patients have already developed immunity against specific AAVs serotypes because of exposure to AAVs, AAVs are no longer suitable for those patients.^[^ ^62^ ^]^ AVs are associated with brisk immune responses. In a trial of OTCD, the use of adenoviral gene therapy caused the death of a patient.^[^ ^63^ ^]^ The safety of viral vectors must be particularly considered in clinic. Moreover, limited packing capacity is a major obstacle for viral‐mediated gene therapy. AAVs can only load 4.5 kb genome and is not suitable for large DNA elements. For widely used in therapeutic genome editing, many studies focus on refining viral vector systems to achieve higher transduction efficiency and better safety profiles. Viral vectors still have a bright prospect for delivering genome editing tools for research and application.^[^ ^64^ ^]^

## 4 DELIVERY OF CAS9:SGRNA RNP

As Cas9 and sgRNA are the core working components of CRISPR, direct delivery of Cas9:sgRNA RNP could be efficient for gene editing. Since SpCas9 (the most widely used endonuclease) is a large and positively charged protein,^[^ ^99^ ^]^ and sgRNA is a short and negatively charged synthetic RNA, the RNP complex can be formed by simply incubating Cas9 and sgRNA at room temperature.^[^ ^72^ ^]^ However, the negatively charged RNP cannot enter into cells and undergo degradation by enzymes. In response to these issues, one approach is to make the RNP more permeable to cell membranes and the nucleus by modifying Cas9 or sgRNA. Another more frequently used method to promote the delivery of RNP is to use carriers. Cationic lipids, polymers, and NPs that can absorb or condense the negatively charged RNP complex through electrostatic interaction have been employed as carriers to protect RNP from degradation and deliver RNP into cells (Table 3). However, efficient RNP delivery is challenging because the intracellular cytoplasm delivery efficiency of RNP is low due to difficult endosome escape and Cas9 nuclease is a relatively large protein ≈160 kDa) that cannot enter the nucleus through nuclear pores. Sometimes, two strategies are used in combination to enhance RNP delivery. It is worth noting that the low charge density of RNP is unfavorable to forming stable NPs constructed by electrostatic interaction. Efficient cytoplasm delivery and RNP entry into the nucleus are prerequisites for highly efficient delivery systems of RNP. This section will review the strategies to achieve efficient RNP delivery and highlight the challenges for clinical applications of CRISPR using RNP.

**TABLE 3:** Delivery of Cas9:sgRNA RNP

Delivery system	Target gene	Genome editing	Cells or organ	Disease	Reference
Lipid NPs					
Lipidoids	GFP	NHEJ	HeLa‐Dsred cells, GFP‐HEK cells	–	^[^ ^65^ ^]^
	GFP, tdTomato	NHEJ	GFP‐HEK cells, HeLa‐DsRed cells, brain	Neurological disorder	^[^ ^66^ ^]^
	GFP	NHEJ	GFP‐HEK cells	–	^[^ ^67^, ^68^ ^]^
	GFP, tdTomato	NHEJ	A549 cells, HeLa cells, mouse brain neurons	–	^[^ ^69^ ^]^
	PD‐L1	NHEJ	Subcutaneous tumor	B16F10‐cancer	^[^ ^70^ ^]^
	PCSK9	NHEJ	Muscle	DMD	^[^ ^71^ ^]^
Lipofectamine	EGFP, CLTA, EMX1, VEGF tdTomato	NHEJ, HDR	EGFP‐U2OS cells, neuron‐derived mouse embryonic stem cells; cochlea	–	^[^ ^72^ ^]^
	tdTomato,	NHEJ	Neural progenitor cells, HEK293T cells, brain	Neurological disorder	^[^ ^73^ ^]^
	GFP, HBB, EMX1, CXCR4, Rosa26, PCSK9	HDR	HEK293T cells, K562 cells, Mouse embryonic stem cells	–	^[^ ^74^ ^]^
Lipofectamine/PCL/polyDOPA‐melanin	GFP	NHEJ	EGFP‐U2OS cells	–	^[^ ^75^ ^]^
Polymeric NPs					
Cationic PEI	mecA	NHEJ	Methicillin‐resistant Staphylococcus aureus	Antimicrobial	^[^ ^76^ ^]^
TransIT‐X2	EMX1, GAA	NHEJ, HDR	HPSCs, HEK293T cells	–	^[^ ^77^ ^]^
PLGA	γ‐globin	NHEJ	HPSCs	–	^[^ ^78^ ^]^
	Cdk5	NHEJ	Subcutaneous tumor	B16F10‐cancer	^[^ ^79^ ^]^
Black phosphorus nanosheets	GFP	NHEJ	Subcutaneous tumor	B16F10‐cancer; CT26‐cancer	^[^ ^80^ ^]^
Gold NPs	AAVS1, PTEN	NHEJ	HeLa cells, macrophages	–	^[^ ^81^ ^]^
	BFP, mutated dystrophin	HDR	BFP‐HEK cells, muscle	DMD	^[^ ^82^ ^]^
	mGluR5, YFP	NHEJ	HEK293T cells, neurons, brain	Fragile X syndrome	^[^ ^83^ ^]^
	CCR5, gamma (γ)‐globin	Cas9/Cpf1‐HDR	HSPCs	HSPCs engraftment	^[^ ^84^ ^]^
Zeolitic imidazolate framework‐8	GFP	NHEJ	GFP‐CHO cells	–	^[^ ^85^ ^]^
Zeolitic Imidazole Framework‐90	GFP	NHEJ	GFP‐HeLa cells	–	^[^ ^86^ ^]^
Mesoporous silica NPs	PCSK9, APOC3, ANGPTL3	NHEJ	Liver	Cardiovascular disease	^[^ ^87^ ^]^
	EGFR	NHEJ	Subcutaneous tumor	H22‐Cancer	^[^ ^88^ ^]^
Gold nanowires	GFP	NHEJ	GFP‐B16F10 cells	–	^[^ ^89^ ^]^
Upconversion NPs/SiO_2_/PEI	PLK‐1	NHEJ	Subcutaneous tumor	A549 cells‐cancer	^[^ ^90^ ^]^
Liposome‐templated hydrogel NPs	PLK‐1	NHEJ	Subcutaneous tumor	U87‐Cancer	^[^ ^91^ ^]^
Macrovesicles	GFP	NHEJ	GFP‐U2OS cells	–	^[^ ^92^ ^]^
DNA Nanoclews	GFP	NHEJ	GFP‐U2OS cells	–	^[^ ^93^ ^]^
DNA linker	EGFP, EMX1	NHEJ	Hela cells, HEK293 cells	–	^[^ ^94^ ^]^
	Hsp90α	NHEJ	A375 cells, HEK293T cells	A375‐Cancer	^[^ ^95^ ^]^
Cell‐penetrating peptide conjugate	CCR5	NHEJ	U87 cells, GS5 cells	–	^[^ ^96^ ^]^
Asialoglycoprotein receptor ligands	EMX1	NHEJ	HEPG2 cells	–	^[^ ^97^ ^]^
Red fluorescent protein/chitosan	PRDX4	HDR	HEK293T; RAW264.7; HeLa; U2OS; A549	–	^[^ ^98^ ^]^

### 4.1 Modifying Cas9 protein to enhance RNP delivery

**FIGURE 3:**
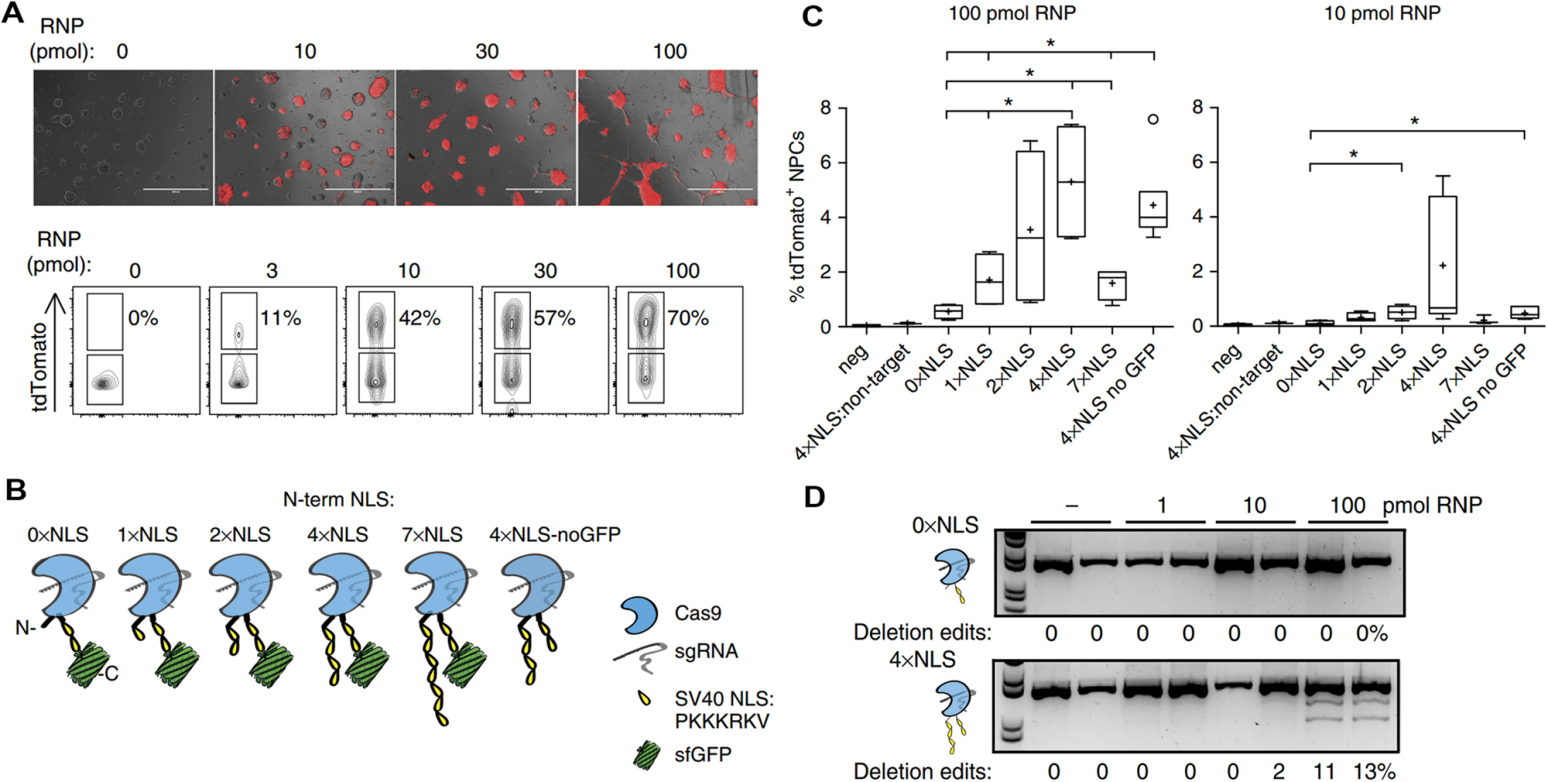
NLS for Cas9 RNP delivery. (A) Gene editing efficiency is RNP dose dependent. (B) N‐terminal 1–7×NLS‐Cas9‐2×NLS design. (C) Direct delivery of 1–7×NLS‐Cas9‐2×NLS with NPCs led to activation of tdTomato reporter in genome‐edited cells. 4×NLS‐Cas9‐2×NLS designs are more efficient at genome‐editing cells than other designs. (D) Genomic DNA PCR of tdTomato stop locus following RNP direct delivery validates tdTomato+ flow cytometry analysis. Reproduced with permission.^[^ ^73^ ^]^ Copyright 2017, Nature Publishing Group

Unfavorable properties (e.g., impermeability into the nucleus) necessitate the modification of Cas9 protein. Cas9 proteins can be modified by common protein engineering methods without compromising its activity, such as conjugation chemistry and recombinant protein technology, to enable carrier‐free targeted delivery of RNP.^[^ ^100^ ^]^ For example, cell‐penetrating peptide (CPP), which are a class of positively charged, short amphiphilic peptides composed of 5–40 residues and can cross the lipid bilayer of eukaryotic cells with minimal damage, have been extensively used to promote cytoplasmic delivery.^[^ ^101^ ^]^ Kim et al. conjugated a recombinant Cas9 protein with a cysteine residue at the C terminus and a maleimide‐linked CPP (sequence: 4G9R4L) to afford a cell membrane permeable Cas9‐m9R, while a positively charged cysteine modified CPP (sequence: C4G9R4LC) was used to complex sgRNA into sgRNA:9R NPs, possibly due to the formation of polymerized CPP through disulfide bond.^[^ ^96^ ^]^ The positively charged Cas9‐m9R and sgRNA:9R NPs were both efficiently taken up by the cells and successfully knocked out the target gene with minimal off‐targeting effects in several human cell types. However, the gene‐editing efficiency was variable and significantly dependent on the cell type. Although complexing cationic Cas9‐m9R and anionic sgRNA is not attempted to form RNP for achieving gene editing, the increased number of positive charges on Cas9 might facilitate the delivery of RNP theoretically. Similarly, transactivator of transcription (TAT) peptide was also applied to conjugate Cas9 protein by a transglutaminase reaction to overcome drug resistance by knocking out the multidrug‐resistant (MDR1) gene. As a result, the TAT‐RNP complex outperformed Lipofectamine 3000 mediated delivery of RNP in MDR1 gene disruption and significantly enhanced doxorubicin's intracellular accumulation and potency in drug‐resistant cells.^[^ ^102^ ^]^ In addition, the Doudna group ligated Cas9 to dendrimers of asialoglycoprotein receptor ligand (ASGPrL, a liver‐specific receptor) via disulfide exchange reaction for targeted delivery of Cas9 RNP into HEPG2 cells.^[^ ^97^ ^]^ Cas9‐ASGPrL RNP can be efficiently internalized by HEPG2 cells mediated by the ASGPr receptor and release the Cas9 in the cytoplasm triggered by the cytosol GSH. Moreover, NLS, a short amino acid sequence, can expand the nuclear pore to 25 nm^[^ ^103^ ^]^ and has been widely exploited to modify large proteins for nucleus delivery. For example, Doudna et al. engineered Cas9 with SV40 derived NLS peptide (sequence: PKKKRKV) at the different terminus and variable numbers and studied its effects on cell uptake and nuclear transport of Cas9 in neural progenitor cells (NPCs) (Figure 3).^[^ ^73^ ^]^ They found that NLS modification on the N‐terminal (nxNLS‐Cas9‐2×NLS) successfully improved the cellular uptake and nuclear entry of RNP and that four NLSs per Cas9 on the N‐terminus (4×NLS‐Cas9‐2×NLS) was optimal.

Synthetic polymers have been conjugated onto Cas9 to enhance the delivery of RNP. For example, Chung et al. used maleimide‐functionalized, branched PEI (*M*_w_ = 2000 and 25,000 Da) to react with the sulfhydryl groups on Cas9 to obtain SpCas9‐bPEI, which formed NPs with sgRNA to display superior transfection efficacy to native RNP in methicillin‐resistant *Staphylococcus aureus*.^[^ ^76^ ^]^ Conjugation of Cas9 with PEI significantly enhanced the RNP uptake than the complex of Cas9 and PEI/lipofectamine, and gene knockout by SpCas9‐bPEI restored the sensitivity of drug‐resistant bacteria to oxacillin. Besides, cationic polymers can also be used to enhance the delivery of native RNP by electrostatic interaction.^[^ ^104^ ^]^ In addition to the modifications mentioned above for carrier‐free transfection of RNP, Liu et al. designed a recombinant Cas9 nuclease protein to render Cas9 negatively charged so that the modified RNP can be delivered into cells by cationic transfection reagents.^[^ ^72^ ^]^

### 4.2 Modifying sgRNA to enhance RNP delivery

Engineering guide RNA through covalent chemical modifications, alterations in the spacer length, and sequence modifications is another important approach to enhance RNP's delivery efficiency^[^ ^100^ ^]^ and reduce off‐target effects and immune response.^[^ ^105^ ^]^ For example, Saha et al. engineered sgRNA with an extended streptavidin binding aptamer loop for modular co‐assembly with biotinylated molecules and streptavidin. They assembled the modified sgRNA, Cas9, and biotinylated single‐stranded oligodeoxynucleotide (ssODN) donor template with streptavidin to obtain an RNP complex for precise HDR.^[^ ^77^ ^]^ As a result, transfection of complexes of RNP and ssODN using TransIT‐X2 significantly improved precise gene editing via HDR. Furthermore, Liu et al. synthesized a polyanionic sgRNA (≈100 phosphate groups) with more charge than the native sgRNA to improve the delivery of Cas9 protein (+22 net theoretical charge) using commercial transfection reagents. This approach achieved an EGFP gene knockout efficiency of 80% in U2OS EGFP reporter cells and a GFP knockout efficiency of 20% in the inner ear hair cells of living Atoh1‐GFP mice.^[^ ^72^ ^]^ Compared to modifications of cationic Cas9, modifications of anionic sgRNA usually reduce the cellular uptake of more negatively charged RNP, requiring the use of additional cationic carriers. Besides, sgRNA may be modified with hydrophobic moieties and polymerizable functional groups like siRNA to enhance the delivery.^[2e^ ^]^

### 4.3 Nanomaterial‐based carriers for Cas9 RNP delivery

Although the modification of Cas9 protein and sgRNA can enhance RNP delivery into the cytoplasm and nucleus in a carrier‐free manner, the modified RNP can still be easily degraded by enzymes, and the activity of RNP may be significantly reduced by improper modification.^[^ ^106^ ^]^ Moreover, Cas9 exposure can elicit strong adverse immune responses. In contrast, RNP delivery by nanomaterial‐based carriers can improve drug stability and targeted delivery and enhance lysosomal escape.

#### 4.3.1 Lipids

Lipids consisting of a polar head group and a hydrophobic tail can be engineered into well‐defined and transformable nanostructures for broad biomedical applications.^[^ ^107^ ^]^ Among various nanoparticulate carriers, lipid‐based NPs, including liposomes, are the most widely studied and have successfully entered the clinic to deliver small‐molecule drugs and nucleic acid (such as siRNA and mRNA).^[^ ^108^ ^]^ Native or modified RNP can be either encapsulated into liposomes or complexed with cationic liposomes for enhancing gene editing efficacy. For example, Liu et al. devised negatively charged recombinant proteins to enable protein delivery using commercial cationic transfection reagents.^[^ ^72^ ^]^ The recombinant Cas9 nuclease protein was designed to render Cas9 negatively charged so that Cas9 was efficiently transfected into cells by Lipofectamine RNAiMAX and Lipofectamine 2000. However, the gene knockout efficacy of recombinant Cas9 RNP was less than that of native RNP, demonstrating the gene knockout efficiency of RNP is highly variable and dependent on the cargos and carriers. It should be noted that the recombinant Cas9 may have reduced activity than native Cas9 and disturbed interaction with sgRNA. Maximizing the delivery efficiency of RNP, Xu et al. synthesized a library of chalcogen‐containing lipids via the Michael addition reaction. They examined the structure‐activity relationship between lipidoids and intracellular protein delivery efficiencies in HeLa cells.^[^ ^65^ ^]^ In vitro screening using negatively charged (–30) GFP‐Cre protein showed that lipidoids with O17Se tail were most efficacious. The distinct performance of lipoids with Se‐derived tails might be ascribed to facilitated endosomal escape. O17Se tailed lipidoids also showed higher transfection efficacy in the lung than O‐ and S‐derived lipidoids after IV injection. Due to the different physicochemical properties of (–30) GFP‐Cre and native RNP, they recommended that RNP‐loaded LNPs should be re‐optimized, which has been confirmed by the fact that native RNP loaded LNPs showed comparable GFP knockout efficacy with native RNP/Lipofectamine 2000 complexes in GFP‐HEK cells. Since RNP must be released from the lipid‐based carriers, lipids with rapid intracellular degradation are desirable. In their previous study, the same group developed a library of bioreducible disulfide‐linked lipidoids to facilitate the cytoplasmic release of RNP.^[^ ^66^ ^]^ Similarly, in vitro studies showed that lipidoids showed superior efficacy in delivering the recombinant negatively charged GFP‐Cre protein than Lipofectamine 2000 but displayed comparable efficacy in the delivery of native RNP. Moreover, Leong and Becker et al. used bioreducible lipid to encapsulate Cas9 RNP to knock out the IL1RAP gene in human leukemia stem cells (LSCs) to inhibit relapse of acute myeloid leukemia.^[^ ^109^ ^]^ LNP‐Cas9 RNP and chemokine CXCL12α were loaded onto scaffolds, which were coated with mesenchymal stem cell membrane‐coated nanofibers to mimic the bone marrow microenvironment. The release of CXCL12α from the scaffolds induced the migration of LSCs to the scaffolds, enabling targeted gene editing of LSCs by LNP‐Cas9 RNP and attenuating LSC growth. Liposome‐templated hydrogel NPs (LHNPs) were developed by the Zhou group to deliver CRISPR/Cas9 for anticancer therapy.^[^ ^91^ ^]^ The core of LHNPs was composed of PEI hydrogel, formed via host‐guest supramolecular assembly. Cas9 and sgRNA expressing minicircle DNA were loaded into the nanogel. The surface of LHNPs was anchored with iRGD ligand for tumor targeting and with mHph3 CPP for enhanced cellular uptake. In vitro genome editing results showed that LHNPs downregulated the expression of the PLK‐1 gene and dramatically suppressed the growth of U87 and GS5 cells. In vivo studies showed that targeted delivery of Cas9/minicircle sgPLK1‐2 using LHNPs successfully edited the PLK‐1 gene in U87 subcutaneous xenograft tumor and intracranial tumor after IV injection and inhibited tumor growth. Although LNPs with different functions (e.g., enhancing lysosomal escape and triggered degradation) have been developed and have shown considerable gene‐editing efficacy in vivo, the gene‐editing efficiency is still far from ideal. In addition, co‐delivery with ssDNA to achieve homology‐directed repair requires more attention.

#### 4.3.2 Cationic polymers

Cationic polymers, which contain a high density of amine groups, have strong interactions with negatively charged cargos, show endosomal escape capability because of the proton sponge effect, and have been widely used to deliver nucleic acid.^[^ ^110^ ^]^ For example, Gu et al. used DNA nanoclews to complex Cas9 RNP via the base‐pairing mechanism to protect RNP from degradation and facilitate the engineering of RNP‐loaded NPs by providing additional negative charges. Positively charged PEI was further complexed with DNA nanoclews/RNP complexes to achieve efficient intracellular delivery and endosomal escape of RNP. In vitro experiment showed that DNA nanoclews enhanced the EGFP knockout efficiency, ascribed to programmable cytoplasm delivery of RNP.^[^ ^93^ ^]^ Similarly, DNA nanoclews were used to assemble Cas12a/crRNA‐PCSK9 and then coated with PEI and a layer of charge reversal polymer bearing galactose as targeting moiety sequentially to afford a nanoparticulate formulation for reducing cholesterol level in serum.^[^ ^111^ ^]^ The nanoparticulate formulation exhibited hepatocyte targeting CRISPR delivery, knocked out PCSK9 with an efficiency of approximately 48%, and successfully regulated the cholesterol levels. Liu et al. complexed the negatively charged red fluorescent protein (RFP) with cationic chitosan (CS) to obtain RFP@CS, and subsequently absorbed Cas RNP and ssDNA to afford Cas9 RNP‐RFP@CS nanoassemblies. To obtain stable Cas9 RNP‐RFP@CS nanoassemblies, they inserted 20 glutamate residues (E‐tag) in the N terminus of the Cas9 protein to enhance electrostatic interactions with the RFP@CS NPs. To improve the efficacy of nucleus entry, they engineered the Cas9 protein with 3×NLSs at the C terminus. Gene editing using RFP@CS NPs achieved 12.5% gene knockin efficiency for GFP gene in HEK293 cells. Additionally, delivery of Cas9 RNP by RFP@CS NPs to target PRDX4 genes in HEK293T, RAW264.7, HeLa, U2OS, and A549 cells also resulted in efficient indel gene editing. Notably, treating cells with RFP@CS at a higher dosage (400 μg/mL) for 24 h showed non‐cytotoxicity.^[^ ^98^ ^]^ In another study, Cheng and Leong et al. reported multifunctional NPs for RNP controlled release.^[^ ^112^ ^]^ A nitrilotriacetic acid decorated NPs were loaded with Ce6 photosensitizer, captured the his‐tagged Cas9 RNP, and coated with a cationic iRGD‐PEGylated polymer to construct multifunctional NPs for integrin‐mediated targeted RNP delivery into tumor cells. Once the multifunctional NPs were internalized by tumor cells, they could escape the endosomes and enter the cytoplasm with the help of reactive oxygen species produced by Ce6 upon NIR irradiation. Eventually, RNP was released by GSH induced cleavage of the disulfide bond to perform gene editing.

#### 4.3.3 Inorganic NPs

**FIGURE 4:**
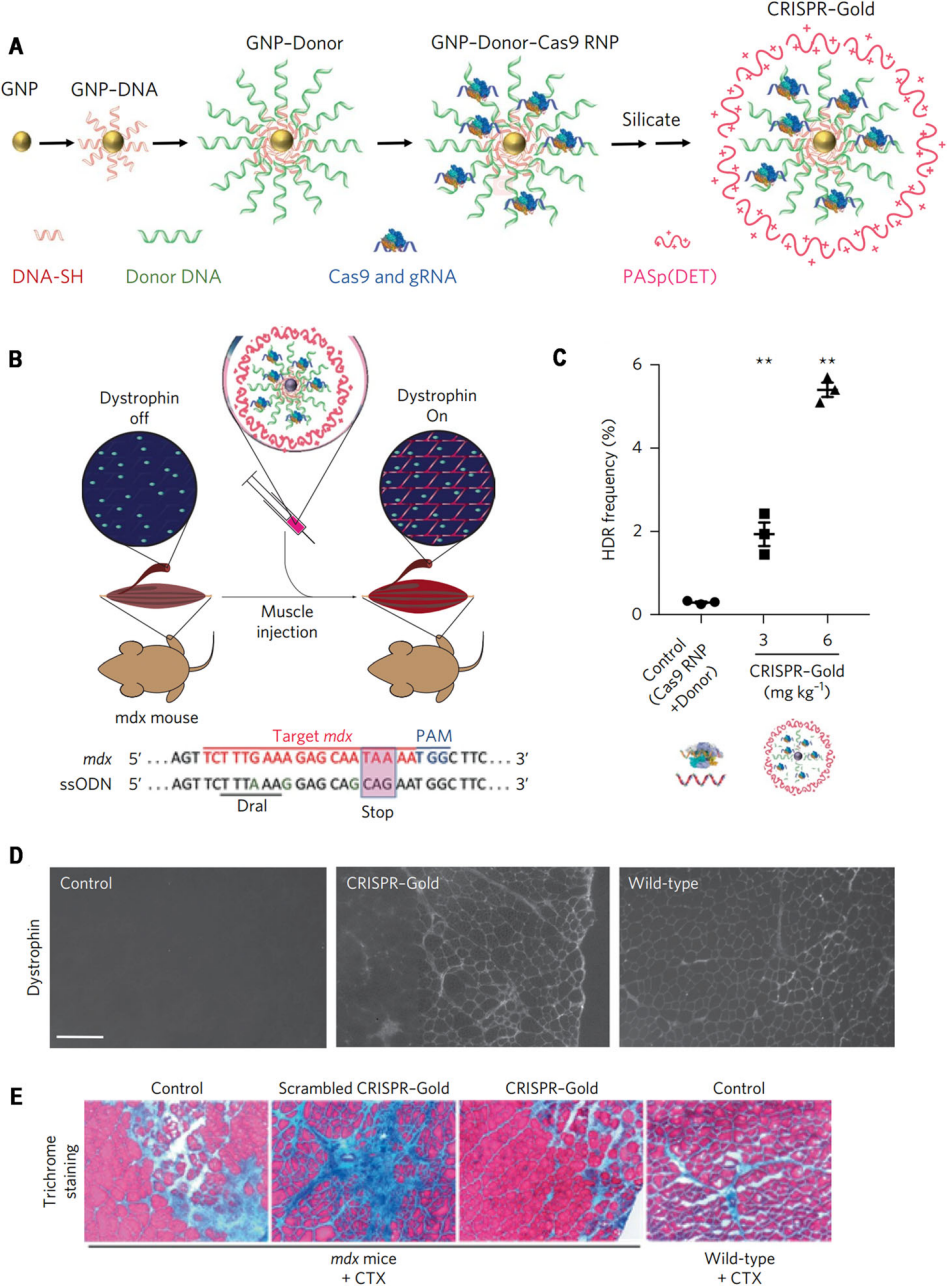
Gold NPs for RNP delivery to promote HDR. (A) Schematic illustration of synthesis of CRISPR‐Gold. (B–E) CRISPR‐gold promotes HDR in the dystrophin gene and dystrophin protein expression, and reduces muscle fibrosis in mdx mice, with CTX stimulation. (B) CRISPR‐Gold was injected into the hind leg muscle of eight‐week‐old mdx mice simultaneously with CTX. Bottom: dystrophin mutation sequence and donor DNA design. The donor DNA sequence designed to repair the nonsense mutation is marked in the pink box. The nucleotides marked in green (A, G and G) are silent mutations that prevent Cas9 activity on the edited sequence. (C) CRISPR‐Gold‐induced genome editing in the dystrophin gene was confirmed by deep sequencing. (D) CRISPR‐Gold‐injected muscle of mdx mice showed dystrophin expression (immunofluorescence), whereas control mdx mice did not express dystrophin protein. (E) CRISPR‐Gold reduces muscle fibrosis in mdx mice. Reproduced with permission.^[^ ^82^ ^]^ Copyright 2017, Nature Publishing Group

Inorganic nanomaterials, such as gold, iron, and silica NPs, possess a high surface‐volume ratio, well‐tunable size and shape, and unique physical properties and have been widely studied in drug delivery.^[^ ^113^ ^]^ Among them, gold‐based nanomaterials can be easily modified with thiol‐containing molecules through the gold‐thiol chemistry and are thus widely used to deliver biomacromolecules.^[^ ^114^ ^]^ For example, Rotello et al. decorated gold NPs with arginine to obtain ArgNPs for delivery of negatively charged RNP. They engineered the N‐terminal of Cas9 with a polyglutamate peptide tag to increase the charge density of RNP and the C‐terminal of Cas9 with NLS protein to promote the nucleus entry of RNP.^[^ ^81^ ^]^ The number of glutamate (E) repeats significantly affected the size and cellular uptake of nanoassemblies of ArgNPs and RNP, and the cytoplasmic delivery efficiency of Cas9En increased as the E‐tag length increased from E0 to E20. Intracellular distribution study indicated the instant release of Cas9E20 from nanoassemblies after endocytosis and efficient accumulation of Cas9E20 in the nucleus. Mechanistic studies showed that nanoassemblies entered cells mainly through a cholesterol‐dependent membrane‐fusion‐like uptake pathway. Gene editing using Cas9E15‐RNP/ArgNPs achieved 30% knockout efficiency for PTEN and AAVS1 genes in HeLa cells. Knockin using CRISPR system have the potential to cure genetic disorders via HDR. In 2017, the Murthy group developed a gold NPs‐based method to deliver Cas9 protein, sgRNA, and donor DNA (ssOND) to correct DMD causing DNA mutation.^[^ ^82^ ^]^ Firstly, thiolated Gold NPs (GNPs) were modified with thiol‐oligonucleotides which have a region complementary to ssOND, and subsequently complexed with RNP using layer‐by‐layer technology (Figure 4). Then, the negative charge density of Cas9 RNP/Donor DNA/GNPs was increased with the incubation of sodium silicate. Finally, CRISPR‐Gold was obtained by complexing silicate‐Cas9 RNP/Donor DNA/GNPs and endosomal disruptive polymer PAsp(DET). CRISPR–Gold was low toxic and induced HDR, showing about 4% HDR efficiency in several cell lines, including hiPSCs, hESCs, and primary myoblasts. CRISPR‐Gold successfully promoted in vivo dystrophin expression via HDR with minimal off‐target effects. This is the first example of HDR in vivo achieved using non‐viral vectors to deliver Cas9, sgRNA, and donor DNA. In another study, the same group coated gold NPs with single‐stranded thiol‐oligonucleotide without sequence specificity and then complexed with Cas9 or Cpf1 RNP, and subsequent coating of a layer of endosomal disruptive PAsp(DET) polymer to afford CRISPR‐Gold.^[^ ^83^ ^]^ Delivery of Cas9 RNP or Cpf1 RNP successfully edited targeted genes in different cell types, with comparable efficiency after intracranial injection in hippocampus and striatum. Therapeutic studies showed that mGluR5‐CRISPR knocked out the expression of mGluR5 gene and rescued mice from increased repetitive behaviors. Similarly, Adair and co‐workers engineered AuNP/CRISPR with layer‐by‐layer conjugation of the Cas9 or Cpf1 nucleases, guide RNA, and single‐stranded DNA (ssDNA) donor template on the surface of AuNP via semi‐covalent gold‐thiol interaction and successfully delivered CRISPR into HSPCs for targeted HDR.^[^ ^84^ ^]^ Gold nanowires (AUNWs), another type of gold‐based nanomaterials, were also employed by the Wang group as ultrasound (US)‐powered motors to deliver RNP.^[^ ^89^ ^]^ The authors immobilized Cas9 protein onto the surface of AUNWs via a disulfide bond. Cas9/sgRNA@AuNWs was rapidly internalized by B16F10 cells with the aid of ultrasound. They found Cas9:sgRNA RNP was released into the cytoplasm by intracellular glutathione (GSH) and efficiently transported into the nucleus, showing up to around 80 % GFP knockout within 2 h of cell incubation. In addition, silica‐based carriers were also studied for improved RNP delivery due to their biocompatibility, porous structure, and ease of surface modification.^[^ ^90^, ^115^ ^]^

#### 4.3.4 2D materials

2D materials are a class of materials whose size is reduced to the limit of atomic layer thickness in one dimension and is relatively large in the other two dimensions. 2D nanomaterials have a lamellar structure that provides a large surface area for high drug loading.^[^ ^116^ ^]^ Among 2D nanomaterials, 2D‐graphene oxide (2D‐GO) can be easily modified due to functional groups on their surface and have emerged as novel drug delivery carriers.^[^ ^117^ ^]^ For instance, Zhou et al. conjugated GO with PEG‐PEI via an amide bond to absorb negatively charged Cas9 RNP. GO‐PEG‐PEI/Cas9 RNP downregulated the gene expression of EGFP in mRNA level and protein level in EGFP‐AGS cells. Gene knockout efficiency of 100 nM GO‐PEG‐PEI/Cas9 RNP in EGFP‐AGS cell was about 39% two days after transfection.^[^ ^118^ ^]^ In addition, Yu et al. utilized black phosphorus nanosheets (BPs) for enhanced cytosolic delivery Cas9:sgRNA RNP.^[^ ^80^ ^]^ In order to facilitate the nucleus transportation and loading by BPs, endonuclease was fused with three repeating NLS at the end of the C terminus. BPs were biodegradable and released 72% of RNP in 12 h in vitro. In MCF‐7 cells, Raman intensity mapping and fluorescence tracking of BPs indicated that fluorescent RNP was released from BPs, escaped from the endosome, and entered nuclear at 12 h after incubation. 78.0% nuclear transporting rate of released RNP was achieved after 24 h. Intratumoral injection of RNP silencing EGFP into A549/EGFP tumor successfully reduced expression of EGFP, showing the potential of BPs for genome editing via NHEJ.^[^ ^80^ ^]^

#### 4.3.5 Zeolitic imidazolate frameworks (ZIFs)

ZIFs are a subfamily of metal‐organic frameworks (MOFs), formed by metal ions (M) and imidazolate anions, with many attractive features, such as high porosity, pH sensitivity, large surface areas, and responsive degradation, enabling them to be used as “smart” drug delivery systems.^[^ ^119^ ^]^ For example, Khashab et al. synthesized positively charged ZIF‐8 to deliver negatively charged RNP through electrostatic interaction.^[^ ^85^ ^]^ The release behavior of Cas9 from ZIF‐8 loaded with Cas9 protein and sgRNA (CC‐ZIFs) was pH‐dependent. Under the neutral condition, ZIF‐8 was stable, while ZIF‐8 was quickly degraded to release Cas9 at pH 6. In vitro studies showed that protonation of the imidazole‐based framework at endosomal pH facilitated Cas9's endosomal escape and enhanced nucleus delivery. In addition, the CC‐ZIFs were non‐toxic to EGFP‐CHO cells and downregulated the gene expression of GFP by 37% over four days. In addition, Mao and Wang et al. developed an ATP‐responsive ZIF‐90 for Cas9 protein delivery.^[^ ^86^ ^]^ The imidazole‐2‐carboxaldehyde and Zn^2+^ with Cas9 could self‐assemble to ZIF‐90/Cas9 NPs and efficiently encapsulate Cas9 protein, while the wrapped Cas9 could be released in the ATP‐concentrated cytosol. The ZIF‐90/Cas9 NPs were first transfected to GFP expressing HeLa cells, and then cells were transfected with Lipofectamine 2000/sgRNA complexes. As a result, a GFP knockout efficiency of up to 40% was achieved. Although ZIFs have shown considerable gene editing efficiencies in vitro, more in vivo evaluations are needed due to the complicated physiological environment.

#### 4.3.6 Nanocapsules

**FIGURE 5:**
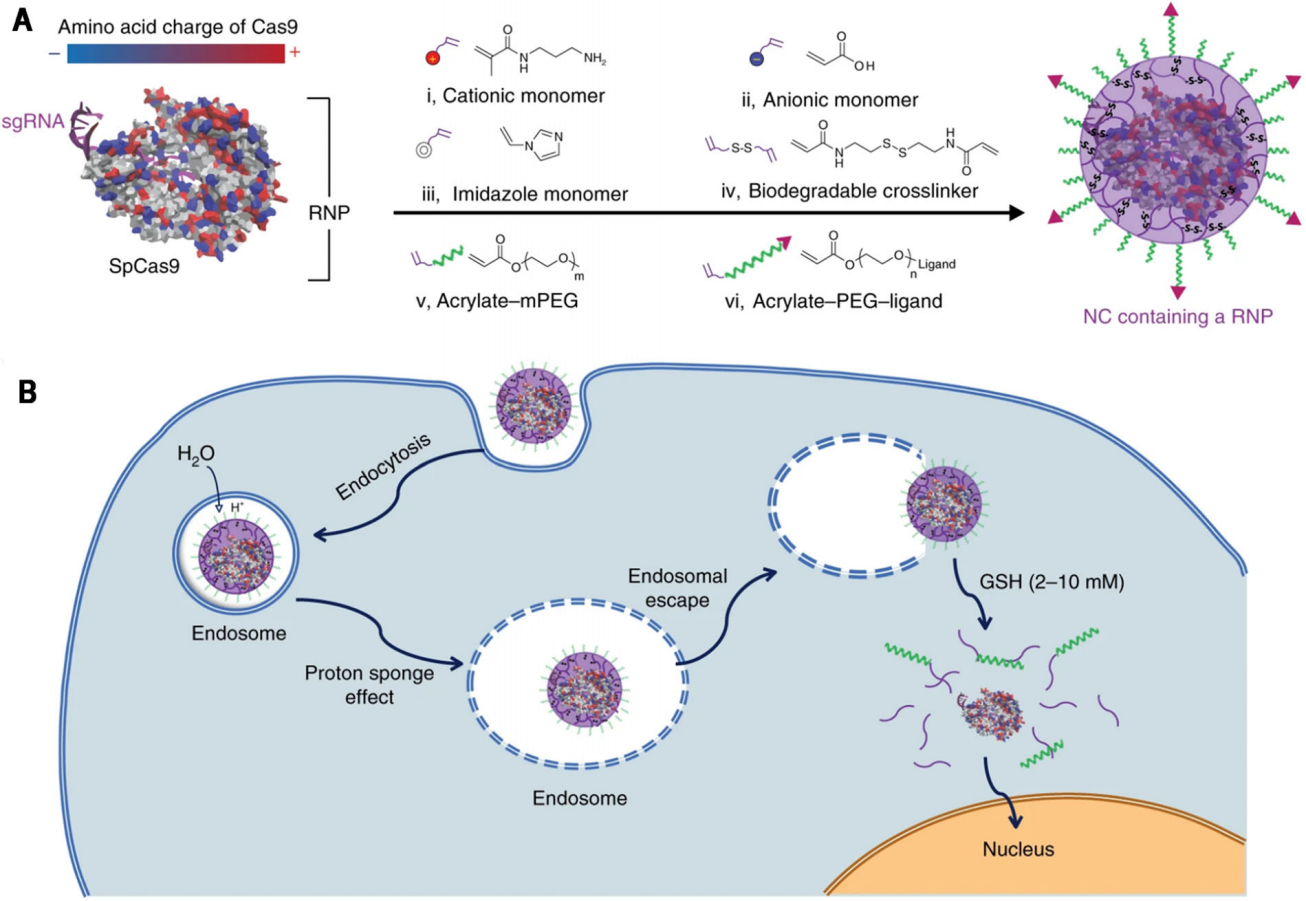
(A) Streptococcus pyogenes Cas9 (SpCas9) has a heterogeneous surface charge due to both positive and negative amino acids residues, as well as the negatively charged sgRNA. A schematic illustration for the formation of the covalently crosslinked, yet intracellularly biodegradable, NC for the delivery of the Cas9 RNP complex prepared by in situ free‐radical polymerization. (B) A schematic depiction of the proposed mechanism of the cellular uptake of NCs and the subcellular release of the RNP. Reproduced with permission.^[^ ^120^ ^]^ Copyright 2019, Nature Publishing Group

Nanocapsules (NCs) represent an important platform to improve the targeted delivery of protein therapeutics with simple non‐covalent encapsulation, featuring small size and reversible protein release profiles.^[^ ^121^ ^]^ The polymer shell of NCs is created by a one‐pot in situ polymerization of monomers on the protein surface and can be designed on demand. Gong et al. used protein Cas9 RNP as a template to synthesize RNP encapsulated NCs for efficient gene editing. Charged, PEGylated, and targeting moiety‐bearing monomers were in situ polymerized with glutathione‐cleavable crosslinker on the surface of RNP by free radical polymerization to afford the customizable NCs (Figure 5).^[^ ^120^ ^]^ The imidazole monomer with pH buffering effect was incorporated to facilitate endosomal escape, and a disulfide crosslinker was used to decapsulate the RNP in the cytosol further. NCs showed higher gene editing efficiency and lower cytotoxicity than lipofectamine/RNP complexes in mCherry‐HEK293 cells and exhibited efficient genome editing in Ai14 reporter mice.

### 4.4 Special issues to be concerned in Cas9:sgRNA RNP delivery

Despite that many nanoparticulate formulations get clinically approved, they are tailor‐made for cargos with specific properties. LNPs have attracted tremendous attention and are considered to have enormous potential to boost gene therapy.^[^ ^122^ ^]^ Nucleic acids with different sequences of similar lengths could be modularly engineered into new, highly efficient formulations based on LNPs for the same targeted tissue. The native RNP is somehow analogous to nucleic acid in molecular weight and charge, which motivates RNP delivery using traditional delivery systems, such as LNP technology.^[^ ^71^, ^107^, ^108^ ^]^ However, delivery systems for RNP are still in their infancy. The delivery of protein complexes (e.g., Cas9:sgRNA RNP) is way more complicated than small molecules and nucleic acids. Although endeavors in screening lipids and modifying Cas9 or sgRNA have been made, the delivery efficiency of RNP is still low and far from ideal. Recent studies showed that some small molecules could stabilize the CRISPR‐related enzyme‐crRNA complex^[^ ^123^ ^]^ or enlarge the nuclear pore to enhance cargo nucleus entry,^[^ ^124^ ^]^ thereby improving gene editing efficiency. These findings should be considered in the development of next‐generation RNP delivery systems. Moreover, at the moment, delivery systems for RNP are primarily evaluated in vitro to silence the targeted gene by inducing mutations via NHEJ, while CRISPR therapies that cure genetic disorders via HDR have been much less studied. For HDR, RNP needs to be co‐delivered with a donor DNA, significantly complicating the development of highly efficient delivery systems, typically showing less than 10% HDR efficiency in vitro. Except for the limitation of low delivery efficiency of RNP and donor DNA, HDR repair is also disturbed by imprecise insertion and deletion of DNA bases.^[^ ^125^ ^]^ In addition, robust immune responses elicited by Cas9 should also be concerned with RNP delivery.^[^ ^126^ ^]^

## 5 DELIVERY OF CAS9 MRNA/SGRNA

Genome editing can also be achieved by delivering Cas9 mRNA and sgRNA (Table 4). Compared to transfection using plasmid, mRNA will afford transient expression of the targeted protein in the cytoplasm, with no risk of integration into the host genome. Nanomaterial‐based carriers can protect the mRNA against nucleases‐mediated degradation and release mRNA into the cytosol after successful escape from endolysosomes and thus are useful in mRNA delivery.^[^ ^138^ ^]^ This section will focus on reviewing lipid‐based delivery systems of Cas9 mRNA/sgRNA, briefly overview other delivery systems, and discuss the concerns for boosting clinical applications of Cas9 mRNA/sgRNA based CRISPR.

**TABLE 4:** Delivery of Cas9 mRNA and sgRNA

Delivery system	Delivery mode of Cas9 mRNA and sgRNA	Target Gene	Disease	Organ	Genome editing	Reference
Lipid‐like NPs	Administered sequentially in separate NPs; mRNA first	HBV gene, PCSK9	HBV	Liver	NHEJ	^[^ ^127^ ^]^
	Administered simultaneously in the same NPs	TTR	Transthyretin amyloidosis	Liver	NHEJ	^[^ ^128^ ^]^
	Administered simultaneously in the same NPs	PLK‐1	Ovarian cancer	Tumor	NHEJ	^[^ ^129^ ^]^
	Administered simultaneously in the same NPs	PCSK9	–	Liver	NHEJ	^[^ ^130^ ^]^
	Administered simultaneously in separate NPs	PCSK9	Hypercholesterolemia	Liver	NHEJ	^[^ ^131^ ^]^
	Administered simultaneously in the same NPs	PCSK9	–	Liver	NHEJ	^[^ ^132^ ^]^
	Administered simultaneously in the same NPs	PTEN	–	Lung	NHEJ	^[^ ^133^ ^]^
	Administered simultaneously in the same NPs	ANGPTL3	Hypercholesterolemia	Liver	NHEJ	^[^ ^134^ ^]^
Dendrimer‐based LNPs	Administered simultaneously in the same NPs	BFP	Nephroblastom	Tumor	HDR	^[^ ^135^ ^]^
Extracellular vesicles	Administered simultaneously in the same NPs Cas9 mRNA and sgRNA were electroporated to RBCEVs, and used them to treat MOLM13 cells	miR‐125	–	–	NHEJ	^[^ ^136^ ^]^
AAVs + Lipid‐like NPs	sgRNA and donor template were delivered by AAVs; Cas9 mRNA was delivered by Lipid‐like NPs	Fah^mut/mut^	Hereditary tyrosinemia	Liver	HDR	^[^ ^137^ ^]^

### 5.1 Lipid‐based carriers of Cas9 mRNA/sgRNA delivery

LNPs, particularly those composed of ionizable lipids with appropriate *p*Ka and endosomal disrupting capability, can efficiently deliver mRNA into the cytosol, where mRNA is translated into functional proteins.^[^ ^139^ ^]^ Recently, lipid‐based delivery systems have successfully entered the clinic to deliver mRNA for vaccination,^[^ ^140^ ^]^ being used to prevent coronavirus disease 2019 (COVID‐19).^[^ ^141^ ^]^ The use of LNPs for non‐gene‐editing mRNA delivery has been extensively reviewed elsewhere.^[^ ^107^, ^142^ ^]^ Here, we mainly review significant advances of lipid‐based carriers in Cas9 mRNA delivery.

**FIGURE 6:**
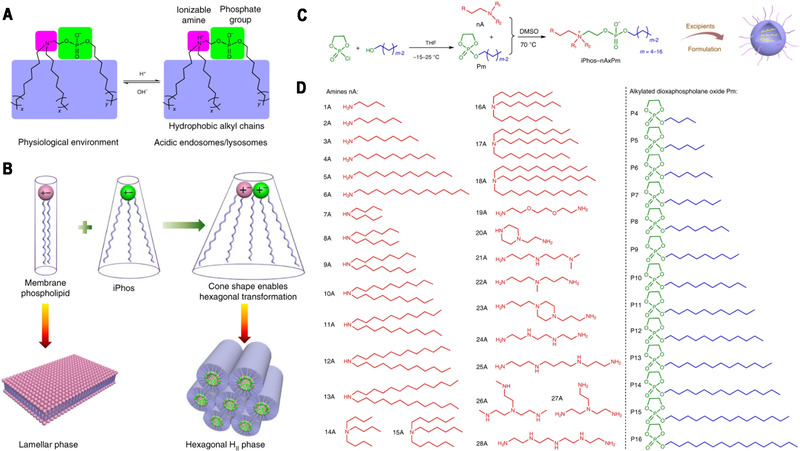
(A) Efficacious iPhos lipids were composed of one ionizable amine, one phosphate group and three hydrophobic alkyl tails. On entering acidic endosomes/lysosomes, protonation of the tertiary amine induced a zwitterionic head group, which could readily insert into membranes. (B) Most biological membrane phospholipids possess a zwitterion and adopt a lamellar phase. When iPhos lipids were mixed and inserted into the endosomal membranes, the formed cone shape by small ion pair head and multiple hydrophobic tails enabled hexagonal transformation. (C) Synthetic routes of iPhos: alkylated dioxaphospholane oxide molecules (Pm) were conjugated to amines (nA) to obtain iPhos (nAxPm). ‘x’ in ‘nAxPm’ indicates the number of Pm molecules modified on one amine molecule. (D) A list of 28 amines and 13 alkylated dioxaphospholane oxide molecules used for iPhos synthesis. Reproduced with permission.^[^ ^133^ ^]^ Copyright 2021, Nature Publishing Group

Anderson et al. combined viral vector and lipid‐based carriers for in vivo HDR gene editing to implement HDR safely and efficiently.^[^ ^137^ ^]^ spCas9 mRNA was encapsulated into LNPs, and a sgRNA expression cassette and an HDR template were packaged into AAVs (termed AAV‐HDR). Transient expression of Cas9 knocked out targeted genes and avoided off‐target genomic damage, while sgRNA/HDR delivered by AAVs maintained the efficacy of HDR. One reason viral vectors are preferred is that they have organ‐homing properties.^[^ ^143^ ^]^ For non‐viral vectors, selectively targeted delivery of CRISPR tools into specific tissues is key to minimizing the off‐targeting effects and maximizing the therapeutic effects. The basic physicochemical properties (such as size, shape, and zeta potential) significantly determine the in vivo fate of NPs, providing a possible strategy to screen targeted delivery systems. For example, well‐tuning the surface charge of lipoplexes led to the systemically targeted delivery of antigen‐expressing RNA into DCs in the spleen for eliciting strong anti‐tumor immune responses.^[^ ^144^ ^]^ Recently, Cheng and Siegwart et al. carefully examined the effects of a supplemental selective organ targeting (SORT) lipid on the traditional liposomes’ in vivo distribution in organs and cell types, providing an unprecedented simple methodology for targeted delivery into lungs, spleens, and livers by manipulating the composition of LNPs.^[^ ^130^ ^]^ They used Cas9 mRNA/sgRNA loaded SORT NPs to exclusively edit extrahepatic tissues. Notably, the supplemental SORT lipid can be further optimized for improving intracellular delivery without losing organ targeting. More recently, the Siegwart group synthesized a series of multi‐tailed ionizable phospholipids (iPhos) to enhance the endosomal escape of LNPs (Figure 6).^[^ ^133^ ^]^ Optimized iPhos lipids composed of one pH‐switchable zwitterion and three hydrophobic tails adopted a cone shape in endosomal environments to facilitate hexagonal membrane transformation and subsequent cargo release from endosomes. Best performing iPhos 9A1P9 formulated LNPs exhibited improved mRNA expression and CRISPR–Cas9 gene editing specificity than traditional lipids, holding promise for treating tissue‐specific diseases. Wang et al. reported reducible lipids composed of disulfide bond‐containing hydrophobic tails for intracellular release of Cas9 mRNA/sgRNA in response to the reductive chemical signals.^[^ ^145^ ^]^ The optimized lipid NPs knocked out the PCSK9 gene by 80% in the mouse. Peer et al. synthesized a library of ionizable amino lipids based on hydrazine, hydroxylamine, and ethanolamine linkers with a linoleic fatty acid chain and an amine head group to enhance the siRNA delivery efficiency.^[^ ^146^ ^]^ They used the optimized amino‐ionizable lipid to deliver Cas9 mRNA and sgRNA for treating cancers.^[^ ^129^ ^]^ In the orthotopic glioblastoma mice model and metastatic ovarian adenocarcinoma mice model, the LNP enabled the in vivo PLK‐1 disruption rate to reach ≈70% and ≈80%, and the survival rate was up to 30% and 80%, respectively. In addition, due to the large size of Cas9 mRNA, the Dong group developed a series of biodegradable amino‐ester lipidlike NPs to improve its delivery. *N*‐methyl‐1,3‐propanediamine (MPA) reacted with 9‐oxononanoic acid (Z)‐non‐2‐en‐yl ester (A) and 9‐oxononanoic acid 2‐ethyl‐hexane‐1‐yl ester (Ab) through a reductive amination reaction to synthesize enzymatically degradable amino‐ester derived lipidlike MPA‐A and MPA‐Ab, respectively. These two lead materials showed a tunable biodegradation rate by adjusting the ester chains and showed a higher delivery efficiency for Cas9 mRNA than the C12‐200 with less toxicity in vitro. In the nude mice bearing xenograft tumors, eGFP signaling was reduced by 41% and 20% after treatment with MPA‐Ab lipidlike NPs and MPA‐A lipidlike NPs.^[^ ^147^ ^]^

Beyond LNPs, cationic lipids have also been used to facilitate the loading of nucleic acids by other types of NPs, such as polymeric NPs. For example, cationic lipid‐assisted NP (CLAN) developed by the Wang group have shown tremendous potential in delivering siRNA, plasmid, and mRNA.^[^ ^148^ ^]^ CLAN loading Cas9 mRNA and guide RNA‐targeting NLRP3 (CLAN_mCas9/gNLRP3_) was comprehensively optimized to target macrophages in vivo for treating multiple inflammatory diseases. CLAN_mCas9/gNLRP3_ successfully ablated the NLRP3 in macrophages to alleviate the acute inflammation in septic shock and peritonitis models, restore insulin sensitivity, and reduce adipose inflammation in a type 2 diabetes model.^[^ ^149^ ^]^

### 5.2 Other delivery systems of Cas9 mRNA/sgRNA

**FIGURE 7:**
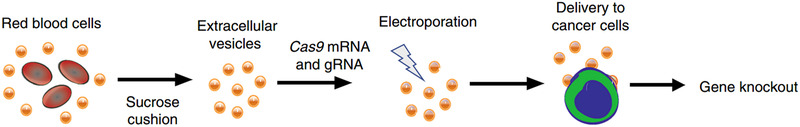
RBCEVs for Cas9 mRNA/sgRNA delivery. Reproduced with permission.^[^ ^136^ ^]^ Copyright 2018, Nature Publishing Group

Other nanomaterial‐based delivery systems, such as extracellular vehicles (EVs),^[^ ^136^ ^]^ nanogels^[^ ^150^ ^]^ and polyplex micelles,^[^ ^151^ ^]^ have also been studied for Cas9 mRNA/sgRNA delivery. For example, EVs, membrane‐related vehicles composed of proteins, lipids, and nucleic acids, have emerged as attractive carriers of RNA due to their inherent biocompatibility and specific signal transmission capacity. Le et al. used sucrose density gradient ultracentrifugation method to isolate EVs from red blood cells (RBCEVs), and then encapsulated cargos by electroporation for efficient and safe delivery (Figure 7).^[^ ^136^ ^]^ RNAs, including antisense oligonucleotides, Cas9 mRNA, and sgRNA, were loaded into RBCEVs separately or in combination. After MOLM13 cells were treated with RBCEVs for two days, about a 90% reduction of targeted gene expression was observed. Endosomal escape is crucial to efficient gene therapy for nucleic acids delivered by NPs. Kataoka et al. synthesized a class of charge reversible cationic poly(*N*′‐(*N*‐(2‐aminoethyl)‐2‐aminoethyl) aspartamide (PAsp(DET)) that can undergo acid‐triggered hydrolysis to become negatively charged. Polycation block copolymer of PAsp(DET) and poly(ethylene glycol) (PEG) condensed Cas9 mRNA/sgRNA into polyplex micelles, which facilitated endosomal escape and cytosol release of Cas9 mRNA/sgRNA in brain cells. Intraparenchymal injection of polyplex micelles deleted tdTomato in neurons, microglia, and astrocytes in the brain of Ai9 mice.^[^ ^151^ ^]^ Eukaryotic genomes are known to contain homologs of the capsid protein of long terminal repeat retrotransposons and retroviruses. Inspired by this, the Zhang group recently reported a modular selective endogenous encapsidation for cellular delivery (SEND) by engineering both mouse and human retrovirus‐like protein PEG10 to deliver RNAs.^[^ ^152^ ^]^ PEG10 can bind specific RNA cargos flanked by PEG10's untranslated regions (UTRs) at UTRs and co‐assemble with a fusogenic envelope protein to form virus‐like particles inside cells. Virus‐like particles packaged with RNAs will be secreted into a culture medium and collected by purification for gene editing. It has been shown that SpCas9 mRNA and sgRNA can be delivered into mammalian cells by SEND. SEND will likely have reduced immune response than the viral vectors and complement existing LNPs. Currently, SEND has demonstrated efficacy at the cellular level, while in vivo pharmacokinetics and targeting ability need to be evaluated.

### 5.3 Special issues to be concerned in Cas9 mRNA delivery

Since Cas9 mRNA needs to edit genes with sgRNA for NHEJ and with sgRNA and donor DNA for HDR, the development of Cas9 mRNA delivery systems is more complex than non‐gene‐editing RNA therapeutics. Although the clinically approved mRNA delivery systems are most likely to be used for Cas9 mRNA delivery, considerable differences in administration routes hamper the development of customized Cas9 delivery systems. The currently approved mRNA‐loaded LNPs are applied by intramuscular injection while treating tissue‐specific diseases with precise gene editing demands systemically administrated nanoparticulate formulations. Systemic delivery systems are more sophisticated than intramuscularly administered mRNA delivery systems, and biological barriers significantly hinder their development. For example, most NPs are majorly cleared by the RES system after intravenous injection and accumulated in the liver and spleen. Besides delivery issues, mRNA can activate pattern recognition receptors in eukaryotic cells, triggering anti‐inflammatory responses. Despite that the immune responses caused by mRNA or lipids may be favorable to activate vaccination, it could be a problem in other scenarios, such as in treating autoimmune diseases.^[^ ^153^ ^]^ Chemical modification of mRNA (such as 5‐methylcytidine (m5C), pseudouridine (Ψ)) is recommended to modulate the immunogenicity without interfering with the translation properties of mRNA^[^ ^154^ ^]^ as well as the mRNA stability.^[^ ^155^ ^]^

## 6 DELIVERY OF PLASMID EXPRESSING CAS9 NUCLEASE AND SGRNA

Delivery of a single plasmid that expresses Cas9 nuclease and sgRNA is the most convenient and cost‐effective strategy for developing CRISPR delivery systems, and developed carriers in gene therapy can be timely applied for this purpose.^[^ ^170^ ^]^ SpCas9 DNA (≈4.2 kb) is one of the most frequently used DNA sequences for packaging plasmid (greater than 9 kb).^[^ ^57^ ^]^ Compared to Cas9 mRNA/sgRNA and RNP, the pCas9 plasmid shows better enzyme tolerance and no immune complications. Plasmids must enter the nucleus to express Cas9 nuclease and sgRNA, and nucleus plasmid delivery is vital for efficient gene editing. Currently, the non‐viral delivery efficiency of plasmids, including pCas9 DNA, is still low. This section will discuss the progress and issues of pCas9 delivery systems (Table 5).

**TABLE 5:** Delivery of plasmid expressing Cas9 nuclease and sgRNA

Delivery systems	Plasmid	Target gene	Cells or disease	Genome editing	Reference
Cationic polymer	Cas9/sgRNA plasmid	HBB, RHBDF1	HeLa cells	NHEJ	^[^ ^156^ ^]^
	Cas9 plasmid, sgRNA plasmid	PLK‐1	A549 cells and Hela cells‐subcutaneous tumor	NHEJ	^[^ ^157^ ^]^
	dCas9/sgRNA plasmid	miR‐524	MDA‐MB‐231 cells‐subcutaneous tumor	–	^[^ ^158^ ^]^
	Cas9/sgRNA plasmid	HPV E7	MDA‐MB‐231 cells‐subcutaneous tumor	NHEJ	^[^ ^159^ ^]^
	Cas9/sgRNA plasmid	VEGFR2	HCC cells‐subcutaneous tumor	NHEJ	^[^ ^160^ ^]^
Liposome	Cas9/sgRNA plasmid	HIF‐1α	BxPC‐3 cells‐subcutaneous tumor	NHEJ	^[^ ^161^ ^]^
Lipid/Inorganic hybrid NPs	Cas9/sgRNA plasmid	PLK‐1	A375‐subcutaneous tumor	NHEJ	^[^ ^162^ ^]^
	Cas9/sgRNA/GFP plasmid	BFP	HEK293	NHEJ, HDR	^[^ ^163^ ^]^
Lipid/polymer hybrid NPs	Cas9/sgRNA plasmid	BCR‐ABL	Chronic myeloid leukemia	NHEJ	^[^ ^164^ ^]^
Polyelectrolyte microcapsules	Cas9/sgRNA plasmid	dTomato	HEK293T	NHEJ	^[^ ^165^ ^]^
Protamine sulfate /CaCO_3_ NPs	Cas9/sgRNA plasmid	EGFP, Luciferase	HEK293T cells, HeLa cells	NHEJ	^[^ ^166^ ^]^
Protamine sulfate/ carboxymethyl chitosan/ KALA/AS1411	Cas9/sgRNA plasmid	CDK11 gene	MCF‐7 cells	NHEJ	^[^ ^167^ ^]^
Protamine/gold nanoclusters	Cas9/sgRNA plasmid	E7	U2OS cells	NHEJ	^[^ ^168^ ^]^
Carbon dots	Cas9/sgRNA plasmid	GFP	HEK 293T cells	NHEJ	^[^ ^169^ ^]^
Semiconducting polymer/PEI/PEG/alkyl side chains/Dexamethasone hybrid NPs	Cas9/sgRNA plasmid	GFP	HCCT 116 cells‐subcutaneous tumor	NHEJ	^[^ ^124^ ^]^

### 6.1 Lipid‐based delivery systems of pCas9

There has been a long history of using cationic liposomes in gene delivery for a variety of applications.^[^ ^171^ ^]^ Quaternary ammonium lipids, such as DOTAP, DOTMA, and DOSPA, have been widely used for in vitro gene transfection and are essential components of many commercial transfection reagents. However, substantial toxicities limit their success in clinical applications. Ideal LNPs for gene delivery should be low toxic and can disrupt the endolysosomal membranes to release cargos into the cytosol. High throughput screening of lipids synthesized by modular chemical reactions, rational design of ionizable lipids with transformable properties in endolysosomes, and surface modifications are predominant strategies to develop efficient lipid‐based gene delivery systems. Optimizations in lipid structure, manufacture processing, and compositions for lipid‐based gene delivery systems can be found elsewhere.^[^ ^172^ ^]^ Here, we mainly review the applications of lipid‐based carriers in pCas9 delivery.

Huang et al. reported an ionizable LNP iLP181 of iLY1809 (p*K*a = 6.43) to deliver psgPLK1 plasmid for treating liver cancer.^[^ ^173^ ^]^ iLP181 showed neutral zeta potential at pH 7.4 and can be protonated in the endosome of acid environment. Protonated lipids can destroy the stability of the endosomal membrane and promote the escape of nucleic acid molecules from the endosome to the cytoplasm. The iLP181 encapsulated psgPLK1 plasmid triggered editing of PLK1 gene with more than 30% in HepG2‐Luc cells and achieved excellent tumor growth suppression without inducing adverse effects. Wang et al. developed pCas9 loaded cationic lipid‐assisted polymeric NPs (CLANs) using PEG_5K_‐PLGA_11K_ and the cationic lipid *N*,*N*‐bis(2‐hydroxyethyl)‐*N*‐methyl‐*N*‐(2‐cholesteryoxycarbonylaminoethyl) ammonium bromide (BHEM‐Chol).^[^ ^164^ ^]^ The efficiency of gene knockout of pCas9‐loaded CLANs was similar to Lipofectamine 2000 and induced 46.8% indel of BCR‐ABL gene in K562 cells after three days. However, the cell toxicity caused by this CLAN is much lower than that of Lipofectamine 2000. Knockout of BCR‐ABL using pCas9‐loaded CLANs significantly reduced gene expression on mRNA and protein level and induced apoptosis of K562 cells. In the CML mice model, CLAN carrying pCas9 prolonged the survival of the CML mice remarkably while inducing low levels of off‐target effects after intravenous injection. In addition, to reduce the off‐target effects of CRISPR/Cas9, the same group constructed CD68 promoter‐driven CRISPR/Cas9 plasmids for macrophage‐specific gene editing. Cas9 plasmid encoding sgNtn1 was efficiently delivered by the CLAN platform to knock out the Ntn1 gene in macrophages and their precursor monocytes in vivo for improved T2D therapy.^[^ ^174^ ^]^ Recently, they used the CLAN platform to co‐deliver autoimmune diabetes‐relevant peptides, pCas9, and three guide RNAs that target costimulatory molecules (CD80, CD86, and CD40) to treat autoimmune diseases.^[^ ^175^ ^]^ This co‐delivery system can downregulate the expression of costimulatory molecules and present the diabetes‐relevant peptide on DCs by MHC class II (MHCII) molecules, inducing immune tolerance for treating autoimmune type 1 diabetes by antigen‐specific Treg cells in mice model. Moreover, Jiang et al. screened out PEG phospholipid‐modified cationic LNPs (PLNP) with the highest transfection efficiency from 56 transfection reagents.^[^ ^176^ ^]^ The PLNP formed a core‐shell structure with plasmid expressing sgPLK‐1/Cas9 nuclease and achieved PLK‐1 knockout efficiency of 47.4% in A375 cells. Intratumoral injection of PLNP/Cas9‐sgPLK‐1a downregulated PLK‐1 related protein expression and inhibited tumor growth in an A375 xenograft tumor model.

In general, delivering plasmid Cas9 by lipid‐based delivery systems is diverse and has shown potential therapeutic effects in mice disease models, such as cancer models and autoimmune disease models. In addition to optimizing lipid delivery systems through library screening, the combination of lipids with other biological materials (e.g., plasma membrane, extracellular vesicles) to improve their targeting ability and safety is an attractive emerging approach. For example, natural nanoscale exosomes, which can transmit multiple signaling molecules and exhibit cell targeting ability due to their particular surface proteins,^[^ ^177^ ^]^ have been used to construct efficient CRISPR delivery vehicles. Tan et al. developed an exosome/liposome hybrid nanoparticulate delivery system to deliver nucleic acid into mesenchymal stem cells (MSCs).^[^ ^178^ ^]^ Vesicle proteins displayed on the surface of exosomes mediated efficient endocytosis of fused NPs into MSCs for achieving efficient gene transfection, leading to significantly reduced mRNA level and the deletion of the target gene in MSCs in vitro.

### 6.2 Cationic polymers‐based delivery systems of pCas9

Through electrostatic interaction, cationic polymers (e.g., chitosan, PEI) can condense negatively charged nucleic acids into small‐sized nanoparticulate polyplexes. The development of highly efficient and safe nucleic acid delivery systems using cationic polymers is still challenging, as the transfection efficacy of polyplexes is affected by several factors, such as molecular weight, charge density, buffering capacity, and amphiphilicity of polymer, and the *N/P* ratio of polymer to nucleic acid.^[^ ^179^ ^]^ Paradoxically, the improved transfection efficacy of polyplexes is often accompanied by adverse issues, such as high toxicity and low cellular uptake. Moreover, the dissociation of weakly bound electrostatic polyplexes caused by the polyelectrolytes in the body leads to the kidney clearance of nucleic acids.^[^ ^180^ ^]^ Polyplexes are often devised with surface shielding, programmable transformation, and biodegradation to improve the in vivo performances.

**FIGURE 8:**
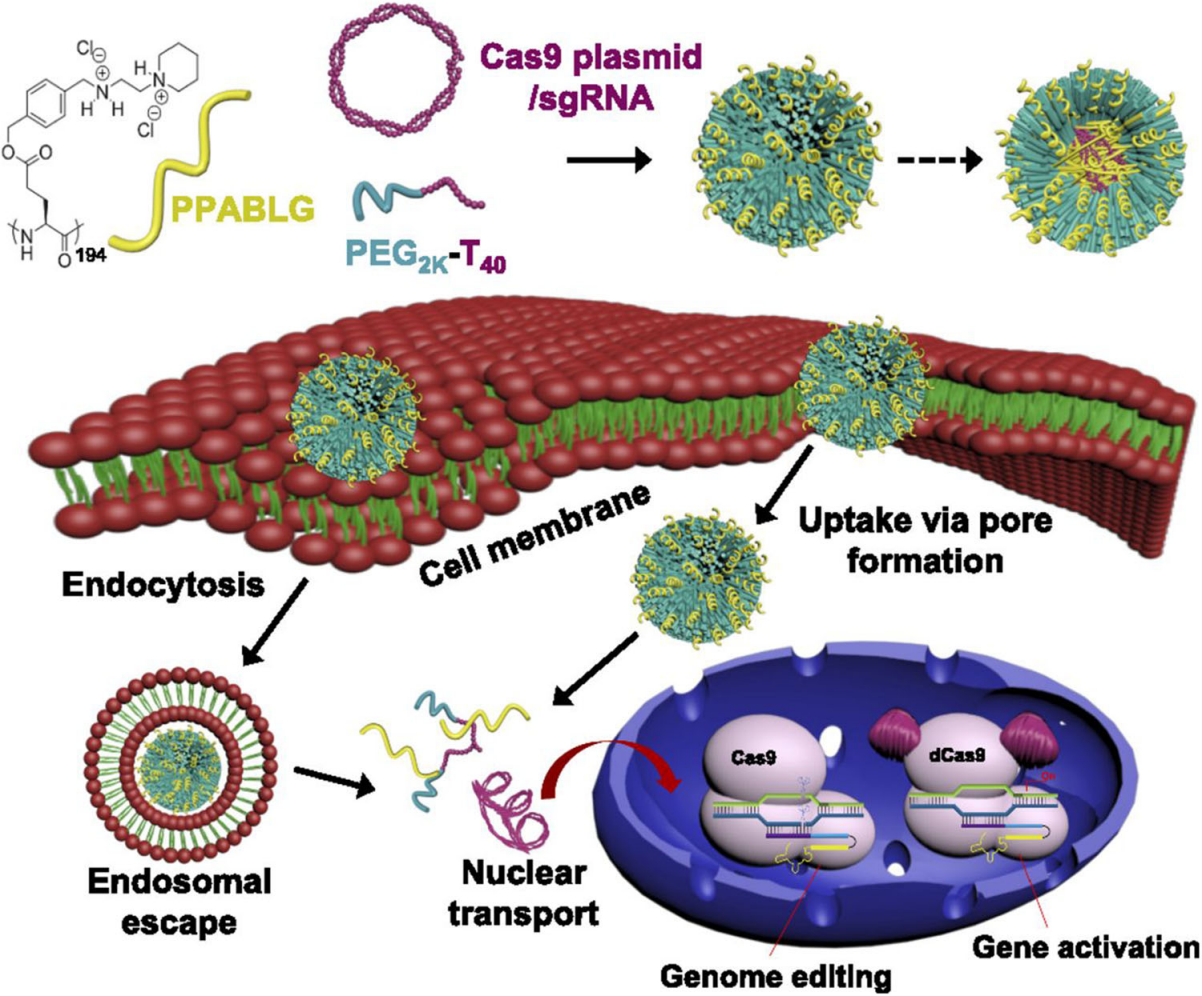
Cationic peptide for Cas9 plasmid delivery. Schematic illustration showing the formation of P‐HNPs and the intracellular activity of Cas9 expression plasmid/sgRNA in performing genome editing or gene activation. Reproduced with permission.^[^ ^157^ ^]^ Copyright 2018, National Academy of Sciences

For example, Huang et al. conjugated low molecular weight PEI onto *β*‐cyclodextrin and complexed PEI‐*β*‐cyclodextrin (PC) with Cas9 plasmid to afford the PEI‐*β*‐CD/Cas9‐sgRNA plasmid nanocomplex via electrostatic interaction.^[^ ^156^ ^]^ The optimized nanocomplex exhibited a high transfection efficiency (≈34%) in Hela cells with moderate toxicity at an *N/P* ratio of 60 and induced 19.1% and 7.0% gene knockout efficiency of HBB and RHBDF1 in Hela cells, significantly higher than that induced by PEI (2.8%). Cationic PPABLG is a polypeptide with a stable helical structure after complexing with nucleic acid and can enhance cargo cellular uptake and endosomal escape in gene delivery. Kam et al. complexed the helical PPABLG with PEG_2k_‐Polythymine_40_, Cas9 expression plasmid, and sgRNA targeting PLK‐1 to form P‐HNPs (Figure 8).^[^ ^157^ ^]^ In the HeLa xenograft tumors model, systemic administration of P‐HNPs loaded with Cas9 plasmid and PLK‐1 targeted sgRNA achieved 35% gene deletion and significantly suppressed the tumor growth. Fluorinated alkyl chains have been shown to facilitate gene transfection and intracellular protein delivery owing to their hydrophobic and lipophobic characteristics.^[^ ^181^ ^]^ Xu et al. synthesized acid‐responsive polycation (ARP) and then modified ARP with fluorinated alkyl chains to produce ARP‐F.^[104b^ ^]^ The delivery of pCas9‐survivin plasmid using ARP‐F exhibited strong tumor inhibition in A549 tumor‐bearing BALB/c nude mice. Notably, the antitumor activity of ARP‐F/pCas9‐survivin was further enhanced when combined with temozolomide treatment by increasing the sensitivity of cancer cells to anticancer drugs.

In cancer treatment, the high expression of immunosuppressive genes (e.g., PD‐L1, CD 47) in tumor cells will reduce the antitumor therapeutic effect.^[^ ^182^ ^]^ The Gong group constructed a series of multifunctional and efficient pCas9 delivery systems by complexing plasmid with a positively‐charged endosomolytic polymer and a negatively‐charged polymer bearing targeting ligand to deplete the immunosuppressive genes of tumor cells.^[^ ^183^ ^]^ For example, they developed a programmable delivery system of CRISPR/Cas9 for efficient tumor immunotherapy.^[^ ^184^ ^]^ Cas9 plasmid simultaneously targeting PD‐L1 and CD47 was first condensed by the fluorinated poly(ethylamine) to form a core, which was subsequently coated with an MMP‐9 cleavable polymer shell to afford the multistage sensitive nanocomplex (MUSE). The shell polymer consisted of hyaluronan backbone, PEG branches, and shielded cell‐penetrating peptide branches. The negatively charged and PEGylated shell enabled the prolonged circulation of MUSE in the blood, whereas the cleavage of MMP‐9 responsive linker between PEG and hyaluronan enabled the exposure of TAT and the hyaluronan backbone, thus facilitating the cellular uptake by CD44‐expressing tumor cells. The endocytosed NPs further underwent lysosomal degradation of hyaluronan to get positively charged and disrupt the lysosomes. After the successful lysosomal escape, the expression of pCas9 depleted PD‐L1 and CD47, co‐activating the innate and adaptive anti‐tumor immunity. Similarly, they developed a delivery system that can respond to enzymes and oxidative stress in the tumor environment to release the Cas9 plasmid, which can deplete the PD‐L1 and protein tyrosine phosphatase N2 genes to achieve highly effective immunotherapy.^[^ ^185^ ^]^

Moreover, Liu et al. constructed a multi‐stage transformable MDNP NP for dCas9–miR‐524 pDNA delivery.^[^ ^158^ ^]^ The core of MDNP is a cationic polyplex of phenylboronic acid (PBA) modified polyethyleneimine (PEI–PBA) and plasmid, while the shell is a layer of negatively charged 2,3‐dimethylmaleic anhydride (DMMA) modified PEG‐*b*‐polylysine. The negatively charged shell and PEGylation could protect MDNP from rapid clearance in the bloodstream. As entering the tumor, the acidic environment disintegrated DMMA groups, leading to exposure of the core and enhancing cell internalization. This multi‐stage delivery strategy achieved high upregulation of miR‐524 and showed efficient tumor growth inhibition in MDA‐MB‐231 tumor‐bearing mice. Aptamer AS1411, a short oligonucleotide sequence obtained by in vitro screening, can target and kill cancer cells, showing the potential for drug delivery.^[^ ^186^ ^]^ Zhuo et al. developed an AS1411 aptamer coupled polymer/inorganic hybrid nanoparticulate delivery system for targeted delivery of pCas9.^[^ ^187^ ^]^ The plasmid was pre‐mixed with calcium chloride and precipitated with sodium carbonate, disodium phosphate, and protamine sulfate (PS). The positively charged nanoparticulate precipitates were then coated with negatively charged biotin and AS1411 aptamer modified carboxymethyl chitosan. This delivery system showed low toxicity and remarkably suppressed CDK11 expression compared to Lipofectamine 2000 and upregulated the expression of the p53 gene in MCF‐7 cells.

### 6.3 Other delivery systems of pCas9

**FIGURE 9:**
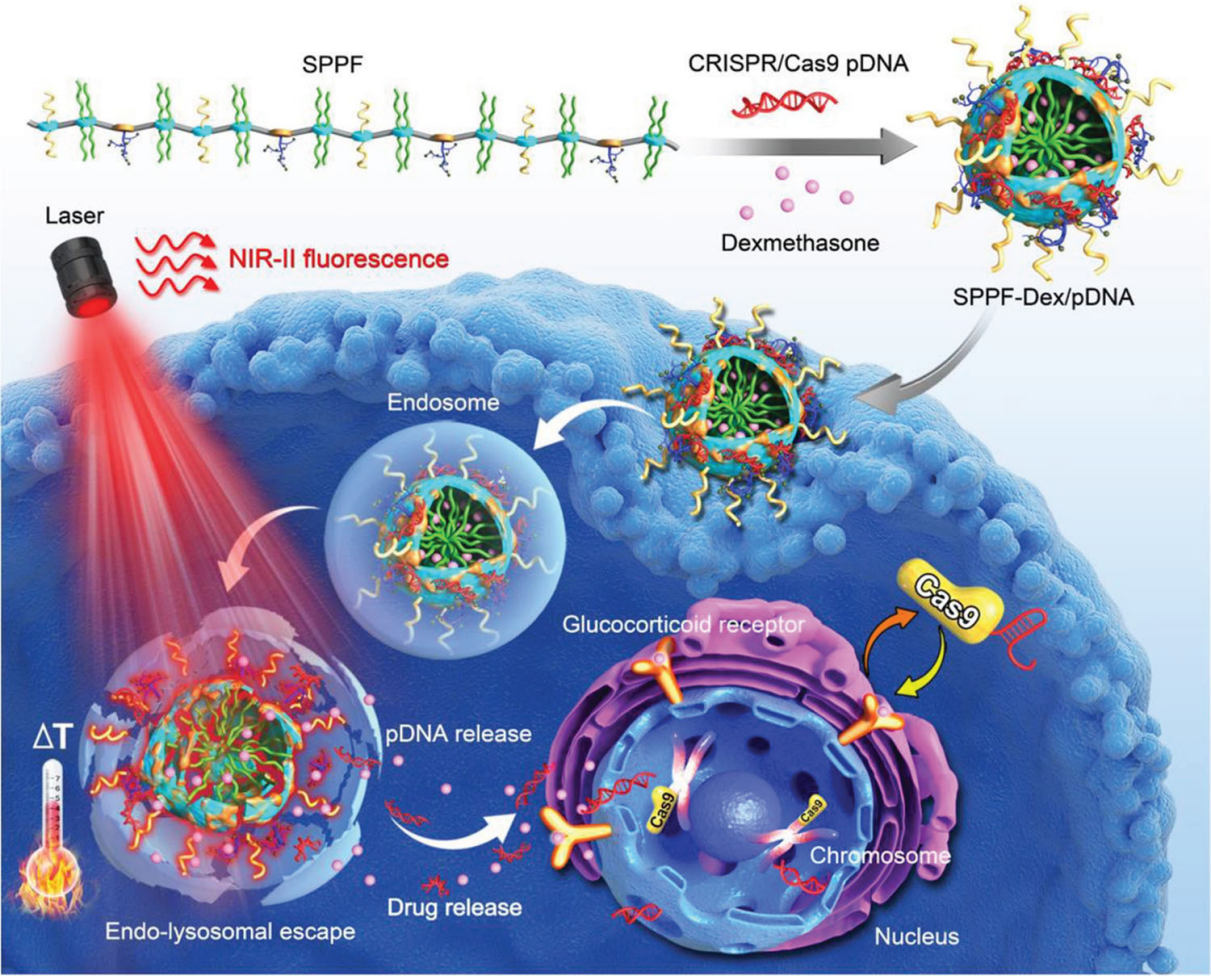
Schematic illustration of SPPF‐Dex NPs design and intracellular genome editing process at 808 nm laser irradiation. Reproduced with permission.^[^ ^124^ ^]^ Copyright 2019, John Wiley & Sons

Besides lipids and cationic polymers‐based delivery systems, many other nanomaterials, such as gold NPs, are also extensively employed for plasmid DNA delivery.^[^ ^188^ ^]^ For example, Jiang et al. used TAT peptide modified gold NPs (AuNPs) to condense Cas9‐sgPLK‐1 plasmid into AuNPs/CP (ACP) for anticancer therapy.^[^ ^162^ ^]^ To prolong the circulation in the blood and facilitate cellular uptake, ACP was further encapsulated into liposomes composed of DOTAP, DOPE, and cholesterol and post‐inserted with mPEG_2000_‐DSPE to form lipid‐coated ACP (LACP). The LACP showed thermo‐sensitive release of plasmid after photoirradiation and knocked out PLK‐1 gene in A375 cells. After systemic administration, LACP significantly suppressed the growth of A375 xenograft tumor with the combination of photoirradiation. Chen et al. modified semiconducting polymers (SPs) with pendant alkyl side chains, PEG chains, and fluorinated polyethylenimine (PF) to synthesize cationic and amphiphilic brush‐structured SPs (SPPF) for co‐delivery of plasmids and dexamethasone (Dex).^[^ ^124^ ^]^ SPPF NPs were prepared and loaded with Dex by a nanoprecipitation method and further complexed with plasmid to obtain SPPF‐Dex/pDNA NPs. Photothermal conversion of SPPF boosted the endosomal escape of NPs and the release of payloads, while Dex bonded to the corticosteroid receptor to enlarge the nuclear pore, promoting the entry of plasmid into the nucleus (Figure 9). In an HCT 116‐GFP mice tumor model, SPPF‐Dex/Cas9‐GFP NPs achieved the GFP gene knockout efficiently, demonstrating the genome editing potential in vivo. Generally speaking, pCas9 is more stable than Cas9 protein and mRNA in the physiological environment, enabling the development of multifunctional delivery systems for combination therapies, such as photothermal therapy, photodynamic therapy, thus improving therapeutic effects.

### 6.4 Special issues to be concerned in delivery of pCas9

One major challenge for pCas9 delivery using non‐viral vectors is the low transfection efficiency. pCas9 must enter the nucleus and be transcribed into Cas9 mRNA. Cas9 mRNA then returns to the cytoplasm and is translated into Cas9 protein, which finally enters the nucleus for genome editing. The complex process and delayed onset decrease the editing efficiency to some extent. In addition, the efficiency of HDR using pCas9 CRISPR is far from being practical, particularly for in vivo HDR.^[^ ^189^ ^]^ Moreover, dsDNA plasmids may randomly integrate into the host genome, and the continuous expression of Cas9 can increase the off‐target effects.

## 7 PROS AND CONS OF THE NANOMATERIAL‐BASED DELIVERY SYSTEMS

We discussed various nanomaterial‐based delivery systems for Cas9 RNP, Cas9 mRNA:sgRNA, and Cas9 plasmid. Each type of delivery systems has its pros and cons. For example, LNPs can be produced in large quantities and are suitable for delivering different format CRISPR tools. They have been successfully used for in vivo delivery of siRNA and mRNA in the clinic. LNPs composed of ionic lipids are engineered under the acidic condition in which ionic lipids are positively charged, whereas LNPs are negatively charged under the physical condition in which ionic lipids are non‐charged. Therefore, LNPs enclose the cargos inside, effectively protecting them from degradation, and are low toxic, but they exhibit efficient lysosomal escape capability. The surface of LNPs can be modulated to endow specific functions, such as avoiding RES clearance and targeted delivery. Nevertheless, LNPs are mainly used for local delivery and some liver‐related diseases after systemic administration due to low systemic delivery efficiency. In addition, cationic lipids may activate the immune response in vivo and have the adjuvant immune effect, so LNPs should be carefully selected for treating different diseases, particularly autoimmune diseases. Cationic polymers can also be used to deliver three different format CRISPR tools by forming polyplexes through electrostatic interactions with cargos. They are often devised to develop programmable intelligent delivery systems to overcome biological barriers in vivo. However, the stability issue is the key for systemic use, as many polyelectrolytes in the body can cause the dissociation or aggregation of polyplexes. Furthermore, the increase of the polymer length results in both high transfection efficiency and toxicity. Gold NPs are also suitable for delivering different format CRISPR tools through either gold‐thiol chemistry or layer‐by‐layer assembly technique. They are a type of inorganic nanomaterials with controllable size, shape, easy surface modification, and unique physicochemical properties and, therefore, can be used for photothermal therapy and ultrasound‐mediated transfection. Silica NPs can be co‐loaded with small molecules and CRISPR tools. CRISPR tools are often loaded using the layer‐by‐layer assembly technique. However, gold NPs and silica NPs are susceptible to exposing CRISPR tools to enzymes, and their safeties should be carefully examined due to slow‐ and non‐degradation. In addition, they may undergo premature release of CRISPR tools in blood circulation like polyplexes.

Emerging CRISPR delivery systems, such as ZIF NPs, DNA nanostructures, and biomembrane‐based NPs, have appealing properties that can improve delivery efficiency. For example, ZIF NPs have a high surface area, adjustable pores, and accessible modification sites, and they can be designed as stimulus‐responsive drug delivery systems. To boost the application of ZIF NPs in the CRISPR delivery, in vivo degradation mechanism needs to be systematically studied. Biocompatible and biodegradable DNA is a kind of intelligent material that can be programmed into different nanostructures and shapes through base pairing and possess dynamic responsiveness and addressability, which can be used for precise drug delivery. However, DNA can be easily degraded in the body. Biomembrane‐based NPs have some membrane proteins of the original cells, so they have natural targeting capabilities compared to traditional liposomes. However, for different diseases, the selection of cell types requires systematic molecular biology research. For the preparation of biomembrane, the separation and extraction process need to be more standardized. In addition, based on allogeneic biomembrane therapy, the issue of immunogenicity requires special attention. Overall, these emerging delivery systems are still in their early stages of development, and a more comprehensive understanding of their advantages and limitations is needed, especially their biosafety and large‐scale production processes need to be further explored.

## 8 THE CLINICAL TRANSLATION OF CRISPR/CAS SYSTEMS

Up to now, there are about 50 clinical trials of CRISPR/Cas systems worldwide. Most of them are adoptive cell therapies, such as CAR‐T therapy, stem cell therapy, to treat blood‐related diseases. Allogeneic adoptive cells are often transfected in vitro by the electroporation method and edited using CRISPR technology to avoid immune rejection. Adoptive T cells are often edited using CRISPR technology to deplete proteins (e.g., PD‐1, and HPK1) that can stifle T cell activation to enhance the engineered T cells' ability to combat blood cancers. Some CRISPR/Cas systems, including the Cas13, Cas12a, and Cas14, show catalytic activity to break non‐specific exogenous nucleic acid sequence after CRISPR/Cas systems recognize the target sequence and thus have emerged as a powerful diagnostic tool to detect viral infection and cancer by lighting the quenched signal on the exogenous nucleic acid sequence. In addition, CRISPR can be used to study the function of epigenetic markers after extracting relevant cells from patient tissues.

Table 6 compiles clinical trials in which viral vectors and nanomaterial carriers are used to deliver CRISPR. For in vitro gene editing, cells are often transiently transfected by the electroporation method. Nevertheless, to permanently maintain the functions of transfected cells, viral vectors are needed. For example, the University of Pennsylvania initiated a clinical trial to treat relapsed or refractory CD19+ leukemia and lymphoma using allogeneic CART19 cells prepared by LVs transduction. So far, only a few trials involve in vivo gene editing, and viral vectors and LNPs are still prevalent. Editas Medicine used AAVs to deliver CRISPR to treat Leber Congenital Amaurosis Type 10 (LCA10) by eliminating the mutation on the CEP290 gene. Excision BioTherapeutics used AAV9 to deliver CRISPR/Cas9 to excise the proviral DNA that HIV integrates into the genome of a human cell to treat HIV‐1‐infection. Intellia Therapeutics initiated two clinical trials (NCT04601051, NCT05120830) to deliver Cas9 mRNA and sgRNA using an LNP platform that can target hepatocytes mediated by ApoE. In the NCT04601051 trial, a single dose of NTLA‐2001 is intravenously administered to treat hereditary transthyretin (TTR) amyloidosis by editing the mutated TTR gene. Phase I clinical trial data indicate that a single dose of NTLA‐2001 reduces serum TTR levels by 87%, and the maximum reduction in TTR on the 28th day was 96%, with no observable side effects.^[^ ^190^ ^]^ Encouraged by these positive results, Cas9 mRNA and sgRNA are delivered by the same delivery platform on clinical trials to treat hereditary angioedema by knocking out the KLKB1 gene in hepatocytes. The First Affiliated Hospital of Sun Yat‐sen University used poloxamer 407 gel to encapsulate CRISPR/Cas9 to treat HPV‐related cervical intraepithelial neoplasia Ⅰ. Shanghai BDgene is using a virus‐like particle‐mRNA platform to deliver Cas9 mRNA to treat refractory viral keratitis by disrupting the gene of HSV‐1 via corneal injection.^[^ ^191^ ^]^

**TABLE 6:** Clinical trials of CRISPR/Cas systems delivered by carriers

Carrier strategies	Diseases	Interventions	Status	NCT number
LVs	Relapsed or refractory CD19+ leukemia and lymphoma	Administer pre‐manufactured allogeneic T cells by eliminating endogenous TCR, HLA‐class I and HLA‐class II	Not yet recruiting; Phase I	NCT05037669
AAVs	LCA10	Eliminate the mutation on the CEP290 gene	Recruiting; Phase I, II	NCT03872479
AAVs	HIV‐1‐infection	Administer intravenously to excise the proviral DNA that HIV integrates into the genome of a human cell	Not yet recruiting; Phase I	NCT05144386
LNPs, Cas9 mRNA and sgRNA	Hereditary transthyretin amyloidosis	Inactivate the TTR gene in hepatocytes	Recruiting; Phase I	NCT04601051
LNPs, Cas9 mRNA and sgRNA	Hereditary angioedema	Knock out the KLKB1 gene in hepatocytes	Recruiting; Phase I, II	NCT05120830
Poloxamer 407 gel, Cas9 plasmid	HPV‐related cervical intraepithelial neoplasia Ⅰ	Knock out the E6/E7 gene of HPV	Unknown; Phase I	NCT03057912
Virus‐like particle‐mRNA, Cas9 mRNA, guide RNA	Refractory viral keratitis	Disrupt the gene of HSV‐1	Active; Phase I, II	NCT04560790

Data listed in this table are derived from https://clinicaltrials.gov/, accessed 2021‐12‐9.

## 9 CONCLUSIONS AND PERSPECTIVES

Significant progress in the field of genome editing has been remarkable in recent years, especially with the emergence of CRISPR, which has dramatically expanded its application. However, there are still many hurdles to be overcome in the clinical translation of CRISPR, such as efficiency issues, safety issues, and specificity issues. Off‐target mutagenesis is a fatal issue in clinical treatment. The induction of oncogenic transformation at the off‐target sites in even the rarest clones (e.g., activating an oncogene) deserves special attention as such change can result in adverse effects. Moreover, off‐target DSBs induced by CRISPR can result in undesired integration of the viral genome. Another major challenge is developing safe and effective CRISPR delivery methodologies. Some viral and nanomaterial‐based carriers could be employed to introduce CRISPR components into target cells. To date, viral vectors are most effective in vivo, especially AAVs, that have been FDA approved to treat inherited disease.^[^ ^50^ ^]^ But pre‐existing immunity to AAVs is prevalent in humans, and constitutive expression of bacteria‐derived Cas9 nuclease by viral vectors could also elicit pathologic immune responses.

To settle these challenges, protein, mRNA, or plasmid delivered by nanomaterial‐based carriers may provide attractive alternatives to viral vectors. In such a way, cargos will be quickly cleared after performing their functions, thereby could minimize nuclease‐induced toxicity and off‐target mutation rate. Different format CRISPR tools have been successfully delivered by nanomaterials for treating various diseases, including cancer and autoimmune diseases, while their delivery efficiencies are still far from ideal. Currently, the state‐of‐the‐art CRISPR delivery systems mainly utilize the NHEJ mechanism to induce mutations to delete target genes, while there are much fewer studies on gene correction by HDR. Nevertheless, we believe that with the development of nanotechnology and biotechnology, CRISPR will provide chances to address some incurable diseases in the future.

## CONFLICT OF INTEREST

The authors declare no conflict of interest.
